# MEF2C Enhances Dopaminergic Neuron Differentiation of Human Embryonic Stem Cells in a Parkinsonian Rat Model

**DOI:** 10.1371/journal.pone.0024027

**Published:** 2011-08-25

**Authors:** Eun-Gyung Cho, Jeffrey D. Zaremba, Scott R. McKercher, Maria Talantova, Shichun Tu, Eliezer Masliah, Shing Fai Chan, Nobuki Nakanishi, Alexey Terskikh, Stuart A. Lipton

**Affiliations:** 1 Del E. Webb Center for Neuroscience, Aging, and Stem Cell Research, Sanford-Burnham Medical Research Institute, La Jolla, California, United States of America; 2 Department of Neurosciences, University of California San Diego, La Jolla, California, United States of America; University of South Florida, United States of America

## Abstract

Human embryonic stem cells (hESCs) can potentially differentiate into any cell type, including dopaminergic neurons to treat Parkinson's disease (PD), but hyperproliferation and tumor formation must be avoided. Accordingly, we use myocyte enhancer factor 2C (MEF2C) as a neurogenic and anti-apoptotic transcription factor to generate neurons from hESC-derived neural stem/progenitor cells (NPCs), thus avoiding hyperproliferation. Here, we report that forced expression of constitutively active MEF2C (MEF2CA) generates significantly greater numbers of neurons with dopaminergic properties *in vitro*. Conversely, RNAi knockdown of MEF2C in NPCs decreases neuronal differentiation and dendritic length. When we inject MEF2CA-programmed NPCs into 6-hydroxydopamine—lesioned Parkinsonian rats *in vivo*, the transplanted cells survive well, differentiate into tyrosine hydroxylase-positive neurons, and improve behavioral deficits to a significantly greater degree than non-programmed cells. The enriched generation of dopaminergic neuronal lineages from hESCs by forced expression of MEF2CA in the proper context may prove valuable in cell-based therapy for CNS disorders such as PD.

## Introduction

Human embryonic stem cells (hESC) are not only an important model system for human neurogenesis [1], [2] but are also a potential source of therapeutic cells to treat neurodegenerative disorders such as Parkinson's disease (PD) [3]–[7], stroke [8], [9], and spinal cord injury [10]–[12]. Such cell-based therapies require protocols for differentiating hESCs into neural precursors and further directing them towards a specialized regional neurotransmitter identity, such as midbrain dopaminergic neurons. However, current methods for culturing hESCs suffer from contamination with animal feeder cells or cells of mesodermal and endodermal origin from embryoid bodies (EB) [13]. Furthermore, hyperproliferation, teratoma formation, and the presence of neuroepithelial cells are commonly observed after transplantation of hESC-derived neural cells into disease models [7], [14], [15]. Hence, *in vitro* induction of hESCs into more restricted neural stem/progenitor cells (hNPCs) prior to transplantation is necessary in order to promulgate therapeutic use in humans. A method of directed differentiation into neurons would facilitate transplantation therapies.

Along these lines, the transcription factor myocyte enhancer factor 2 (MEF2) is a central regulator of gene expression in skeletal and heart muscle [16], neural crest, lymphocyte development, vascular integrity [17], and, as we and others have previously shown, neuronal differentiation and survival [18]–[21]. Among MEF2 family genes in the developing central nervous system (CNS) [22], [23], only MEF2C, initially discovered in our laboratory, is expressed in a tissue- and region-restricted pattern [20], [24]–[26]. Recently, we and others discovered that MEF2C influences NPC differentiation and maturation into neurons during embryonic development [19], and facilitates plasticity by negatively regulating synaptic number and function in mature rodents *in vivo* [27]. Negative regulation of synaptic morphogenesis has also been demonstrated by deleting MEF2A or MEF2D in hippocampal neuronal cultures and rat cerebellar brain slices [21], [28]. Furthermore, our previous report demonstrated that MEF2C directs the differentiation of mouse ESC-derived neural precursors into neurons and suppresses glial fates [18]. In addition to this putative instructive role for neurogenesis, we found that MEF2C promotes cell survival during neuronal differentiation [29].

Concerning differentiation of hESCs into dopaminergic (DA) neurons, a simple *cis*-regulatory element called the DA motif has recently been reported that binds ETS transcription factors and controls the expression of virtually all DA-neuron related genes [30]. In the present study, we noted that there were multiple MEF2 consensus sites in the promoter of a prominent ETS-family member, Etv1. Similarly, we found that an important downstream transcription factor involved in the DA phenotype, nuclear receptor related 1 (nurr1) [31], [32], also contained several MEF2 binding sites in its promoter region. These observations suggested to us that MEF2C might be involved in DA neurogenesis. Unlike Etv1 or nurr1, however, MEF2C also manifests anti-apoptotic, neuroprotective properties, which could prove useful in keeping transplanted stem cells alive [29]. Heretofore little was known about the role of MEF2C in human neurogenesis in general and in DA neuron development in particular.

Here, we report a feeder- and animal factor-free, sphere-based, rosette isolation protocol to derive the neural cell lineage from hESCs with high efficiency and purity. Using these cells, we demonstrate the ability of MEF2C to drive neurogenesis *in vitro* using shRNAs directed at MEF2C or overexpression of a constitutively active MEF2C (MEF2CA) transgene. Further, we show the regulatory role of MEF2C *in vivo* in directing differentiation of DA neurons and the therapeutic potential of hESC-derived NPCs engineered with MEF2CA in a rat model of PD.

## Results

### Feeder-Free Differentiation of hESCs into the Neural Lineage

**Figure 1:**
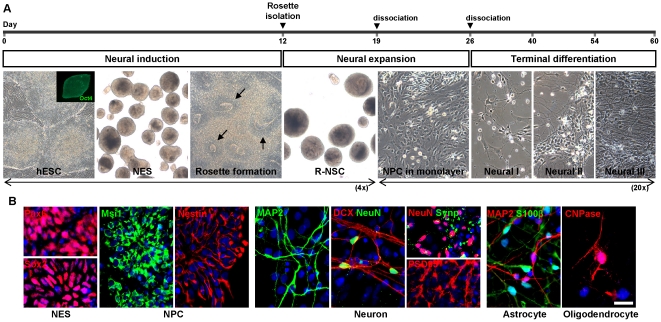
Neural differentiation of human embryonic stem cells (hESCs). (A) Phase-contrast images were taken during various stages of differentiation *in vitro* and represent cell morphology of each developmental step. Arrows indicate rosettes that formed on laminin-coated dishes. hESC, human embryonic stem cell; NES, neuroectodermal sphere; R-NSC, rosette-neural stem cell; NPC, neural progenitor cell (see Materials and Methods). (B) Representative cells from the NES, NPC, Neural I, Neural II, and Neural III stages of development were fixed and stained with the following lineage-specific antibodies. For NPCs: Msi1 (musashi 1) and Nestin. For neurons: DCX, MAP2, NeuN, Synp (synaptophysin), and PSD95. For astrocytes: S100β. For oligodendrocytes: CNPase. DNA stained with DAPI (blue). Scale bar: 25 µm.

H9 hESCs were passaged by manual microdissection and monitored for normal karyotype [33]. hESCs were maintained on Hs27 human fibroblasts but changed to feeder-free conditions when differentiated to facilitate cell-based therapies in humans [34]. To obtain high purity hNPCs derived from hESCs (>95% nestin positive), we established an efficient differentiation method using a feeder-free, neurosphere-based protocol in N2/B27 medium to isolate rosettes (Figure 1A; see Methods for detailed protocols). We verified the differentiation of these cells into the various neural lineages by immunocytochemistry (Figure 1B) and quantitative RT-PCR (qPCR, Figure S1A and S1B). Specifically, from hESCs (Figure 1A inset, Oct4+), we derived neuroectodermal spheres (NES), which harbor rosettes that stain for nuclear Pax6 and Sox2 (Figure 1B). To increase the purity and homogeneity of cells leading to various neural lineages, NES were allowed to attach to the substrate, and rosettes were visualized prior to mechanical isolation. We subsequently dissociated and plated these rosettes, which are known to contain neural stem cells (R-NSCs) [1], [35], [36], [37], [38], to allow them to develop into homogeneous NPCs in monolayer cultures (Figure 1A), as evidenced by their expression of nuclear and cytoplasmic Musashi1 and cytoplasmic nestin (Figure 1B). These NPCs (designated hESC-NPCs) were dissociated and replated. Based on our observations monitoring differentiation of these cells, we divided development during this final plating into Neural Stage I (1 to 14 days post plating), Neural Stage II (15 to 28 days post plating), and Neural Stage III (>28 days post plating). This protocol produced the various neural lineages (Figure 1B), corresponding temporally to normal development *in vivo*. We found that neurons differentiated first, beginning in Neural Stage I (as evidenced by immunostaining for doublecortin (DCX) and microtubule associated protein-2 (MAP2). During Neural Stage II, we observed more mature neurons, as evidenced by staining for NeuN, synaptophysin, and postsynaptic density protein 95 (PSD95). By Neural Stage III, neuronal processes displayed clustering of PSD95 with Synapsin I, suggesting the formation of functional synaptic contacts (Figure S1C). In this final stage, we also found evidence for the differentiation of astrocytes (S100β+ cells), and finally oligodendrocytes (2′,3′-cyclic nucleotide 3′-phosphodiesterase (CNPase)+ cells). Thus, our protocol produced all three neural lineages from highly homogenous and expandable R-NSCs/NPCs.

### MEF2 Proteins Show Differential Expression during Neural Differentiation from hESCs

**Figure 2:**
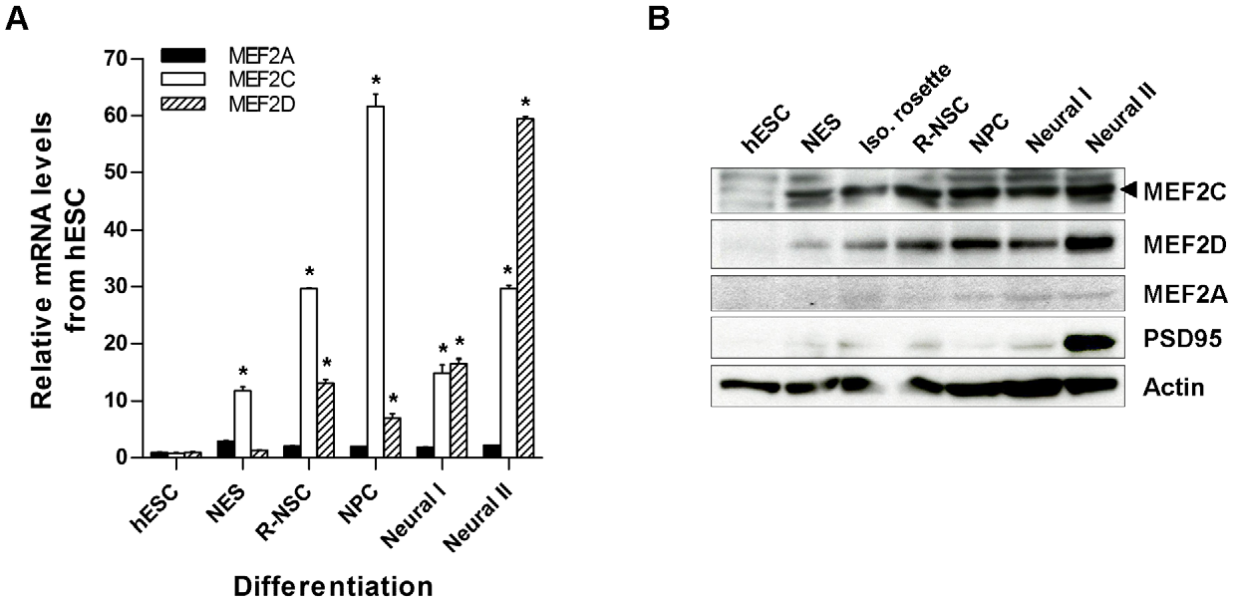
Endogenous expression of MEF2 gene products during neural differentiation. Total RNA or cell lysate was prepared from cells at the hESC, NES, isolated rosette, R-NSC, NPC monolayer, Neural I, or Neural II stages for quantitative RT-PCR (A) or immunoblot analysis (B). Arrowhead indicates endogenous MEF2C. Anti-PSD95 antibody was used to assess neuronal differentiation. Histogram values are mean + SEM, *n* = 3; *p<0.001 compared to hESC by ANOVA.

To begin to dissect the role of the various isoforms of MEF2 during neural differentiation of hESCs *in vitro*, we performed qPCR at various time points (Figure 2A). Expression of MEF2C message was detected at low levels in hESCs. As neural differentiation proceeded, the expression of MEF2C gradually increased through the NPC stage, declined as neurons began to appear (Neural Stage I), and thereafter increased again. Compared to MEF2C expression, MEF2D increased later, beginning at the R-NSC stage, and then more dramatically in mature neurons (Neural Stage II). Though widely expressed in adult brain, expression of MEF2A remained relatively quite low during our differentiation protocol. These data are consistent with the notion that MEF2C is potentially important early in neurogenesis from hESCs.

We next examined the relative protein expression by immunoblot analysis of the MEF2 family of transcription factors during differentiation (Figure 2B). The neuronal postsynaptic protein PSD95 was highly expressed in mature neurons (Neural Stage II), and was hence used as a putative marker for this stage. We detected MEF2C and MEF2D proteins at the NES stage, with a gradual increase through development of NPCs. MEF2C showed relatively higher expression in R-NSCs and NPCs, while MEF2D showed the highest level later at Neural Stage II. MEF2A protein was barely detectable. Protein expression closely matched that of mRNA as assessed by qPCR.

### Knockdown of MEF2C at the R-NSC Stage Causes Decline or Delay in Neurogenesis

**Figure 3:**
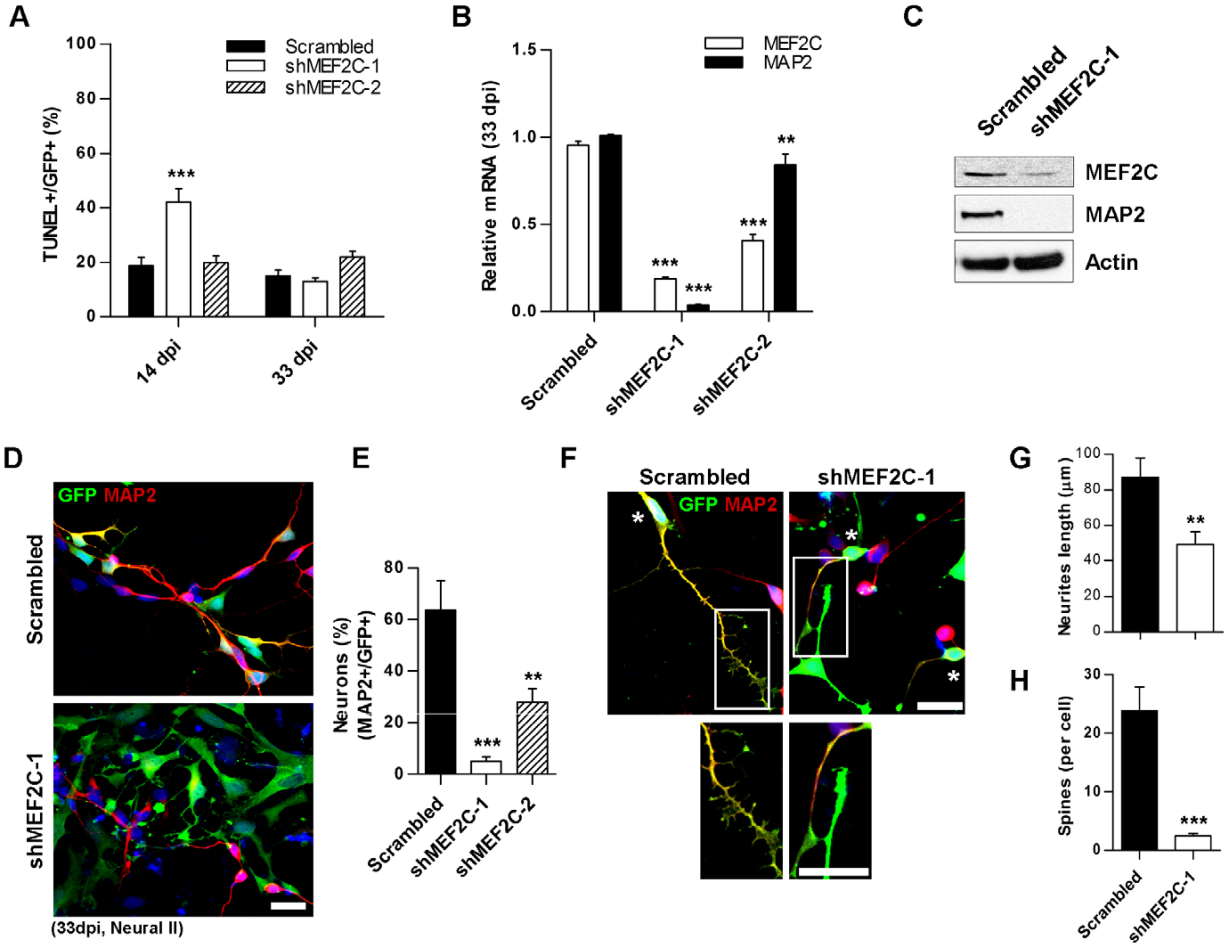
Negative effect of MEF2C knockdown on neurogenesis. (A) TUNEL assay on NPCs at 14 dpi and on Neural Stage II cells at 33 dpi with control or lenti-shMEF2C shRNAs. Lenti-shMEF2C-1-infected cells show increased apoptotic cell death at 14 but not 33 dpi. Values are mean + SEM, *n* = 6; ***p<0.001 by ANOVA. dpi: days post infection. (B, C) Endogenous MEF2C and MAP2 expression at 33 dpi by qPCR and immunoblot. Values are mean + SEM, *n* = 3; **p<0.01, ***p<0.001 by ANOVA. (D) Fewer MAP2+ (red) neurons after lenti-MEF2C-1 shRNA versus scrambled shRNA (GFP+/green) at 33 dpi indicate absent or delayed neurogenesis at Neural Stage II. (E) Percentage of MEF2C shRNA-transduced R-NSCs (total GFP+ cells) differentiated into neurons (MAP2+/GFP+ cells). Values are mean + SEM, *n*≥140 cells scored for each condition; **p<0.01, ***p<0.001 by ANOVA. (F) Shorter dendrites, fewer dendritic spines in lenti-shMEF2C-1-infected cells versus scrambled control. shRNA-infected cells are GFP+ (green), neuronal dendrites are MAP2+ (red); co-labeling (yellow) indicated by asterisks; dendrites in boxes magnified to show spines. DAPI (blue). Scale bars: 25 µm. (G) Dendritic length using Neuron J software (dendrites scored if ≥2-fold longer than diameter of cell body). Values are mean + SEM, *n* = 32 cells; **p = 0.003 by *t*-test. (H) Numbers of dendritic spines for lenti-MEF2C-1 shRNA-transduced neurons and scrambled control (spines scored along entire length of dendrite). Values are mean + SEM, *n* = 17 cells; ***p<0.0001 by *t*-test.

Because we had found that MEF2C expression was highest in hNPCs in monolayer cultures, we hypothesized that MEF2C might be causal in triggering neurogenesis at this stage (an effect distinct from the reported effect of MEF2 transcription factors on synapse formation by mature neurons) [21], [28]. To test this premise, we introduced lenti-shRNAs against MEF2C (lenti-shMEF2C) into R-NSCs prior to the development of NPCs. Each lenti-shMEF2C construct was validated using a target reporter containing the coding region and 3′ UTR of MEF2C. Lenti-shMEF2C-1 exhibited stronger suppression of MEF2C than lenti-shMEF2C-2 (Figure S2A and S2B). Given that MEF2C is also a known cell-survival factor [29], [39] and that smaller-than-normal neurospheres were observed after infection with lenti-shMEF2C-1 virus (Figure S2C), we initially quantified cell death using the TUNEL assay at different time points after lenti-infection. At 14 days post infection (dpi, during the NPC stage), lenti-shMEF2C-1—infected cells displayed an ∼2-fold higher level of cell death than scrambled control-infected cells or lenti-shMEF2C-2—infected cells (Figure 3A), consistent with an early effect of MEF2C on cell viability. However, by 33 dpi (during Neural Stage II), the degree of cell death after lenti-shMEF2C-1 infection was no different than with the other constructs.

As an index of neurogenesis, we therefore examined expression of MEF2C and the neuronal marker MAP2 at 33 dpi when cell viability was stable. We found a significant reduction in both MEF2C and MAP2 mRNA expression and protein levels after infection with lenti-shMEF2Cs (Figure 3B and 3C). Next, we examined neurogenesis and dendritic morphogenesis after MEF2C knockdown. Scrambled control-infected cells manifested substantial numbers of MAP2+ neurons under conditions fostering terminal differentiation, while the vast majority of lenti-shMEF2C-1-infected cells did not show neuronal morphology (Figure 3D). Quantitative assessment of these results showed that MAP2+ neurons, co-stained with GFP to indicate lenti-infection, underwent far less neuronal differentiation if the cells were infected with lenti-shMEF2C-1- or -2 compared to control infection (Figure 3E). As expected since shMEF2C-1 is more effective than shMEF2C-2 in knocking down MEF2C, we observed a dose-dependent effect of knocking down MEF2C with regard to inhibiting neurogenesis. Additionally, scrambled control-infected cells displayed long dendritic processes and clearly-defined dendritic spines, whereas lenti-shMEF2C-1-infected neurons manifested shortened dendrites and a virtual lack of spines (Figure 3F, boxed regions magnified below), as verified by quantitative assessment (Figure 3G and 3H). These data are consistent with the notion that MEF2C is required for the full program of neuronal differentiation and maturation from hESC-derived R-NSC/NPCs.

### Constitutively Active MEF2C (MEF2CA) Increases Neurogenesis in hESC-Derived R-NSC/NPCs

**Figure 4:**
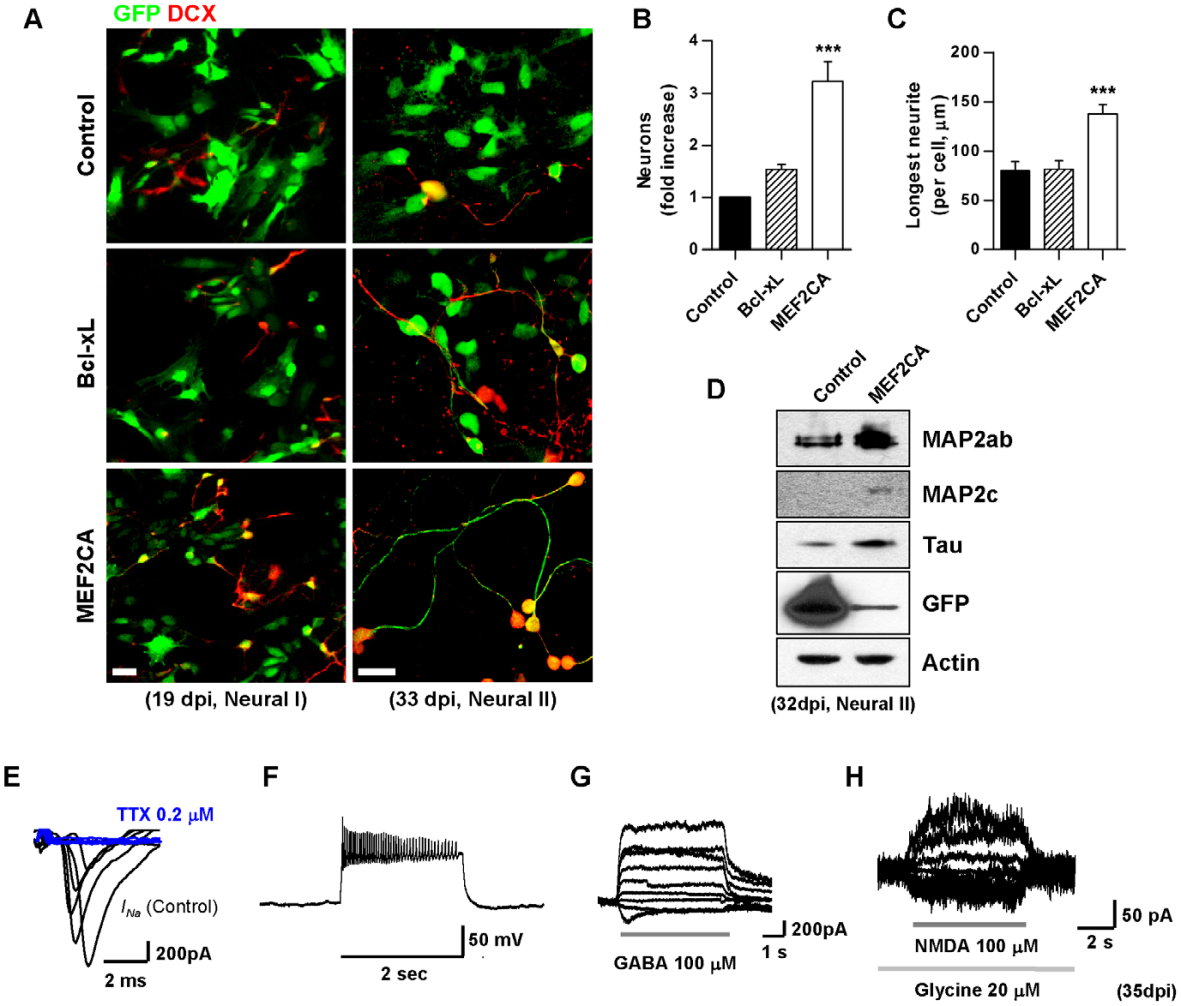
Enrichment in neuronal markers and electrophysiological activity of cells derived from MEF2CA-expressing R-NSC/NPCs. (A) Lentiviral-infected cells plated on poly-l-ornithine/laminin. Anti-GFP antibody identifies infected cells and anti-DCX antibody identifies early neurons. Scale bars: 25 µm. (B) Neuronal enrichment engendered by MEF2CA (*n* = 4 experiments; scheme for infection and analysis shown in Figure S3D). In each experiment, ∼300 GFP-positive cells were scored. Values are mean + SEM, *n*≥1200 cells counted; ***p<0.001 by ANOVA. (C) Longest neuronal process per cell at 33 dpi during Neural Stage II, measured with Neuron J software. Values are mean + SEM, *n* = 61; ***p<0.0001 by ANOVA. (D) Neuronal-specific proteins in lenti-MEF2CA-infected cells by immunoblot during Neural Stage II. Note the much stronger GFP expression in control cells because GFP was expressed from a single gene construct rather than in tandem with MEF2CA and IRES [53]. MEF2CA increased expression of neuronal proteins compared to control infection. (E) Whole-cell recordings of lenti-MEF2CA-infected cells with patch electrodes revealed sodium currents evoked by 100 ms depolarizing steps from −60 to +80 mV in 20 mV increments following 300 ms prepulse to −90 mV (*n* = 5); these currents were inhibited by tetrodotoxin (TTX). (F) Application of 10 pA current steps resulted in depolarization and generation of a “train” of action potentials during current-clamp recordings in *n* = 3 of 8 (37.5%) MEF2CA-infected cells. (G, H) GABA-evoked currents were observed in *n* = 5 of 7 cells (71.4%) and NMDA-evoked currents in *n* = 3 of 3 cells recorded (100%).

Genetic manipulation of transcription factors has been suggested previously as a strategy to generate sufficient numbers of appropriately directed hESC-derived cells for transplantation [40], [41]. In a prior report, we showed that murine ESC-derived NPCs differentiate virtually exclusively into neurons and are protected from apoptosis when stably transfected with MEF2CA [18]. Therefore, we asked if MEF2CA expression would also direct or accelerate neurogenesis from NPC-derived hESCs and increase survival of the neurons thus generated. For this purpose, using our *in vitro* protocol we transduced cells at the R-NSC stage with control or lenti-MEF2CA viral vectors (Figure S3A-S3C), placed the infected cells under terminal neural differentiation conditions, and examined the cells at four subsequent time points (Figure S3D). As a further control, R-NSCs were transduced with the anti-apoptotic construct lenti-Bcl-xL to allow us to distinguish between the pro-survival and neurogenic functions of MEF2C. Our infection efficiency in these experiments was 35–45% and not statistically different among the various test groups, as determined by anti-GFP antibody staining (Figure S3E). We initially examined the effect of MEF2CA on neuronal differentiation by co-staining with neuronal-specific anti-DCX antibody (Figure 4A), and we found that the MEF2CA-infected cells produced 3.2-fold as many neurons as the control groups by 32–35 dpi (during Neural Stage II; Figure 4B). Additionally, these lenti-MEF2CA—infected cells manifested long dendritic processes (Figure 4A, right-hand panel), with a 1.7-fold increase in mean dendritic length over control or Bcl-xL—expressing neurons (Figure 4C). For comparison, we obtained similar effects on human fetal brain-derived neural progenitors infected with lenti-MEF2CA (4.4-fold increase in neuronal marker expression and 2.8-fold longer dendritic processes; Figure S4A–S4D). We further evaluated the effect of MEF2CA on hESC-derived NPCs by examining the level of several neuronal-specific proteins. Compared to controls, lenti-MEF2CA-infected cells manifested increased levels of MAP2ab, MAP2c, and tau (Figure 4D).

To characterize the functional capabilities of neurons derived from lenti-MEF2CA expression in R-NSCs, we made electrophysiological recordings on cells five to nine weeks after infection (Neural Stage III). During whole-cell recordings with patch electrodes, these MEF2CA-programmed cells manifested tetrodotoxin-sensitive sodium currents under voltage clamp (Figure 4E). During current-clamp recording, application of a 10 pA current step resulted in depolarization and generation of a “train” of action potentials (Figure 4F). Additionally, these neurons displayed ligand-gated currents evoked by GABA (Figure 4G) or NMDA plus co-agonist glycine (Figure 4H). These data indicate that the MEF2CA-programmed neurons were electrophysiologically functional and could respond to neurotransmitters.

### MEF2CA Increases DA Neuron-Specific Markers *In Vitro*

**Figure 5:**
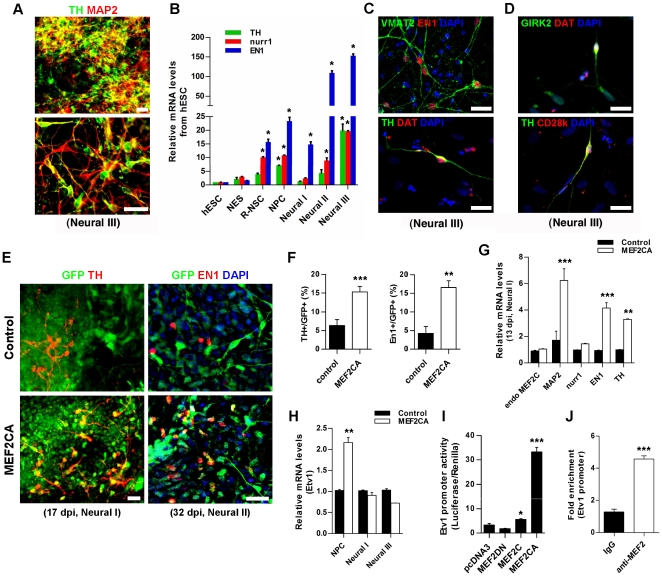
Dopaminergic characteristics of control vs. MEF2CA-expressing hESC-derived neuronal cells. (A) hESC-derived cells at Neural Stage III stained for MAP2 (red/neurons), tyrosine hydroxylase (TH/green, DA phenotype; lower panel magnified). Some TH+ cells did not express MAP2 [54]. Scale bars: 25 µm. (B) Relative mRNA levels of DA-specific genes at various stages. Values: mean + SEM, *n* = 3; *p<0.05 by ANOVA. EN1, engrailed-1. (C) EN1+ or TH+ cells expressed VMAT2 (vesicle monoamine transporter 2) and DAT (dopamine transporter). Scale bars: 25 µm. (D) Expression of GIRK2/DAT+ and CD28k/TH+ cells. (E) Control vector- and MEF2CA-infected cells (GFP+/green) stained for TH (red, left-hand panels) or EN1 (red, right-hand panels), with double-labeled cells (yellow) predominating in the MEF2CA group. Scale bars: 50 µm for TH; 25 µm for EN1. (F) Percentage of MEF2CA/R-NSCs vs. control cells (GFP+) differentiated to DA neurons during Neural Stage I at 17 dpi (TH+, left graph) and Neural Stage II at 32 dpi (EN1+, right graph). Values: mean + SEM, *n*≥1200 TH cells, *n*≥500 EN1 cells; **p = 0.001, ***p = 0.0007 by *t*-test. (G) qPCR shows relative amount of DA gene expression during Neural Stage I at 13 dpi. Values: mean + SEM, *n* = 3; **p<0.01, ***p<0.001 by ANOVA; endo MEF2C: endogenous MEF2C. (H) qPCR shows expression of Etv1 at different stages. Values: mean + SEM, *n* = 3; **p<0.01 by ANOVA. (I) MEF2C activation of 2.1 kb Etv1 promoter-luciferase construct in HeLa cells. Values: mean + SEM, *n* = 6; *p<0.05, ***p<0.001 by ANOVA. MEF2DN: dominant negative MEF2C; MEF2C: full-length MEF2C; MEF2CA: constitutively active MEF2C. (J) ChIP analysis of MEF2C association with the Etv1 promoter. Values: mean + SEM, *n* = 3; ***p<0.001 by *t*-test.

In the absence of exogenous MEF2CA, we found that our protocol for differentiating R-NSC/NPCs resulted in increased expression of endogenous MEF2C (Figure 2A) in addition to some degree of expression of TH+ dopaminergic neuronal markers *in vitro* (Figure 5A). For example, DA neuron-related mRNAs encoding nurr1, engrailed-1 (EN1) and TH began to increase at the R-NSC stage (Figure 5B). With further development, hESC/NPC-derived TH+ neurons achieved a degree of functional maturation in culture, as evidenced by their expression of vesicle monoamine transporter 2 (VMAT2) and dopamine transporter (DAT), although the expression of DAT was weak and observed only in a minority of cells (Figure 5C). Additionally, during neuronal maturation we found evidence at both the message and protein levels for expression of G-protein-gated inwardly rectifying K^+^ channels (GIRK2) and calbindin-D28k (CD28k), which are known to be present in DA/TH+ neurons in the midbrain substantia nigra (A9) and ventral tegmental area (A10), respectively (Figures 5D and S5A) [42]. Under our culture conditions, by Neural Stage III 54.6±3.0% of the MAP2+ cells were also TH+, a comparable or somewhat higher proportion than obtained with previously published protocols [6], [7], [43].

Because the level of endogenous MEF2C expression in R-NCS/NPCs correlated with the subsequent development of DA markers, we next tested if overexpression of MEF2CA would further enhance the DA phenotype. We found that infection with lenti-MEF2CA significantly increased the expression of these DA neuron-related genes and the proportion of anti-EN1/TH—labeled neurons. For example, during Neural Stage I at 17 dpi (Figure 5E, left-hand panels), we observed that lenti-MEF2CA infection yielded 2.4-fold more TH+ neurons than control-infected cells (Figure 5F, left graph). During Neural Stage II at 32 dpi (Figure 5E, right-hand panels), the proportion of EN1+ cells increased approximately four-fold in lenti-MEF2CA—infected cells compared to control infection (Figure 5F, right graph). To further characterize the effect of MEF2CA, we performed qPCR for DA-related gene products (Figure 5G). During Neural Stage I at 13 dpi, MAP2 expression was increased in lenti-MEF2CA—infected cells by 6.2-fold, and EN1 and TH mRNAs were upregulated by 4.1- and 3.2-fold, respectively. By Neural Stage III at 40 dpi, lenti-MEF2CA-infected cells also manifested an increase in nurr1 expression (Figure S5B); endogenous MEF2C increased as well, probably because of the presence of multiple MEF2 sites in its own promoter (Figure S5C). Taken together, these data indicate that feeder-free/neurosphere-based neural differentiation of hESCs can generate DA neurons spontaneously, and MEF2C expression can significantly enrich this process.

Next, we characterized the molecular pathway whereby MEF2 drives the DA phenotype. The activity of ETS transcription factors has been associated with the DA phenotype [30], [44]. Interestingly, we noted that Etv1, an ETS factor proven to be related to DA neurogenesis in the mouse brain *in vivo* [30], has several MEF2 binding sites in its enhancer/promoter (Figure S5C **)**. Hence, we investigated whether MEF2C can increase Etv1 transcription, accounting, at least in part, for the involvement of MEF2C in DA neurogenesis. We found that Etv1 expression increased 2.1-fold in NPCs after lenti-MEF2CA transduction, but not in cells at Neural Stage I or Neural Stage III (Figure 5H). This finding suggests that MEF2C affects Etv1 at an early time point. Therefore, we examined if MEF2C directly regulates Etv1 expression. When MEF2C constructs were transiently co-expressed with an Etv1 promoter-driven luciferase reporter construct, we observed that full-length MEF2C and MEF2CA increased Etv1 expression by 1.7- and 10-fold, respectively (Figure 5I). We then confirmed the direct association of MEF2C with the Etv1 promoter region by chromatin immunoprecipitation (ChIP, Figure 5J). Additionally, we noted that the promoters of several other DA-related genes, including nurr1, EN1 and TH, all possess consensus MEF2 binding motifs (Figure S5C). We found that MEF2C bound to the nurr1 promoter and dramatically up-regulated nurr1 expression at the transcriptional level (Figure S5D and S5E).

### Parkinsonian Rats Transplanted with MEF2CA-Expressing R-NSCs Manifest Improved Motor Function

**Figure 6:**
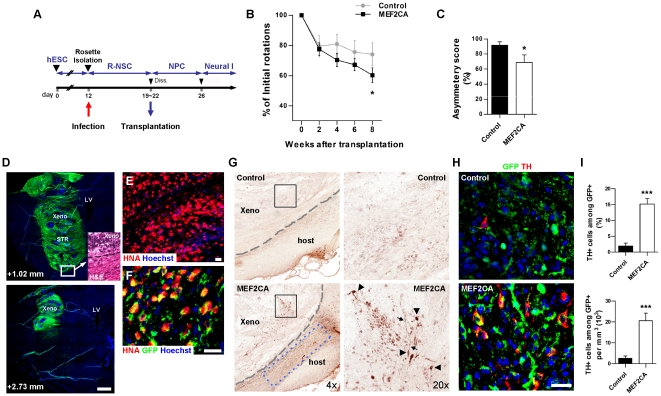
Functional recovery of 6-OHDA-lesioned rats and generation of DA neurons after MEF2CA-derived R-NSC transplants. (A) Infection/transplantation scheme (cf. Fig. 1A). (B) Apomorphine-induced rotations in 6-OHDA-lesionsed rats after R-NSC transplantation into the striatum. Values are mean + SEM, *n* = 14. Rats receiving MEF2CA/R-NSCs show increasing improvement versus controls (*p≤0.035 by ANOVA with planned comparisons post-hoc test). (C) Cylinder asymmetry test 9 weeks post-transplant. Forelimb use in transparent cylinder for 10 min. For score calculation, see Text S1. Values are mean + SEM, *n* = 11; *p<0.03 by *t*-test. (D) Transplanted control R-NSCs (GFP+/green); sagittal brain sections along mediolateral axis. Hoechst dye-stained DNA (blue). Scale bar: 1 mm. Inset: Hematoxylin/eosin (H&E) stain shows xenograft/host boundary (dashed line). Xeno: xenograft; STR: striatum; LV: lateral ventricle. (E) Human origin of cells verified by human nuclear antigen (HNA). Scale bar: 25 µm. (F) Infected cells (GFP+) co-expressing HNA (red) to yield merged yellow fluorescence (remaining GFP positivity represents cell debris). Scale bar: 25 µm. (G) Density of TH+ neurons in MEF2CA/R-NSC xenografts (xeno) close to the ventral area of the striatum was higher than for control/R-NSCs (boxed areas enlarged in right panels). Arrowheads: TH+ neuronal cell bodies; arrows: neuronal processes; dashed lines: graft/host boundary based on H&E staining; blue dashed box: outlines endogenous host TH+ fibers (quantified in Figure S7). (H) MEF2CA/R-NSC versus control/R-NSC DA/TH+ neurons. Transplanted cells are GFP+; TH+ cells are red. Scale bar: 25 µm. (I) TH+ neurons in grafts as percent of GFP+ cells (upper panel). Density of TH+/GFP+ cells in grafts as absolute cell number (lower panel). Values are mean + SEM, *n* = 19; ***p<0.0001 by *t*-test.

Since we found that MEF2C drives the development of the DA phenotype *in vitro*, we next asked if hESC-derived cells that had been programmed with MEF2CA would prove more beneficial *in vivo* than unprogrammed hESC-derived cells when used in a transplantation paradigm to improve motoric function in a rat model of PD. For this purpose, we injected MEF2CA-expressing R-NSCs into the striatum in an attempt to improve behavioral deficits due to 6-hydroxydopamine (6-OHDA)-induced lesions. hESC-derived R-NSCs, prepared as described above, were first dissociated and infected with control- or lenti-MEF2CA viral constructs (Figure 6A). The infected R-NSCs were then grown for 7–10 days as nestin/musashi1-positive neurospheres and dissociated just prior to transplantation. Aliquots of the neurospheres were plated onto dishes to evaluate infection efficiency. Under our conditions, control- and lenti-MEF2CA/R-NSCs displayed 75.6 ± 4.6% and 66.9 ± 5.3% GFP+ cells, respectively (Figure S6A and SB). The fact that infection efficiency was less than 100% in this paradigm was useful because uninfected cells served as an internal control in the transplantation experiments.

The ipsilateral striatum of 6-OHDA-lesioned rats was implanted with 500,000 control lentiviral- or lenti-MEF2CA—infected R-NSCs (*n* = 14). At various time points, starting two weeks after transplantation, the rats were challenged with apomorphine to induce rotation for behavioral/motor testing. Over time, rats receiving control-infected/R-NSCs exhibited a decrease in apomorphine-induced rotations compared with pre-transplantation values (Figure 6B, Control). Notably, with time rats transplanted with MEF2CA/R-NSCs exhibited an increasing reduction in the number of apomorphine-induced rotations compared to rats transplanted with control stem cells, an effect that reached statistical significance by eight weeks (*p*≤0.035; Figure 6B). Therefore, axial function significantly improved in Parkinsonian rats after transplantation of lenti-MEF2CA/R-NSCs compared to control-infected R-NSCs. We also conducted a second motor test to confirm these findings. In this case, the ‘cylinder asymmetry paw use test’ [45] was performed 9 weeks post transplantation to measure forelimb preference during vertical exploration. Lenti-MEF2CA/R-NSC—transplanted rats exhibited a significantly lower asymmetry score compared to control-infected R-NSCs (*p*<0.03; Figure 6C). Hence, rats transplanted with MEF2CA/R-NSCs showed significantly less preference for use of the paw on the non-lesioned side than control rats. Together, these behavioral paradigms indicate that the PD rats receiving MEF2CA/R-NSCs were significantly improved compared to controls.

### Engrafted MEF2CA-Derived R-NSCs Exhibit Increased Survival and Greater Numbers of DA Neurons in Parkinsonian Rats

Next, we sought histological evidence for improvement after transplantation of lenti-MEF2CA/R-NSCs vs. control-infected R-NSCs. After neurobehavioral testing, four rats were chosen at random from each group, sacrificed, and their brains analyzed in detail for survival, integration, and differentiation of the transplanted cells. After transplantation of control-infected/R-NSCs, we observed extensive engraftment into the host rat brain, as shown by the presence of GFP+ cells (Figure 6D). Compared to host cells, the engrafted cells appeared smaller in size with a more irregular arrangement upon hematoxylin and eosin staining (Figure 6D, inset). Under epifluorescence microscopy, the identity of the transplanted cells was verified by the presence of human nuclear antigen (HNA) and expression of GFP (Figure 6E and 6F).

We next examined if MEF2CA-programmed vs. control R-NSCs could generate neurons of DA phenotype *in vivo* after transplantation. First, we examined the grafts by staining with anti-TH antibody using peroxidase immunohistochemistry, and we observed more TH+ cell bodies and processes in the MEF2CA/R-NSC—transplants vs. control-transplants (Figure 6G). Not only were there more TH+ cells in the MEF2CA/R-NSC group, consistent with the notion that the implanted stem cells had become DA neurons, but there were also more TH+ fibers in the adjacent host tissue (Figure 6G, blue box, and Figure S7). Since no GFP+ fibers (representing transplanted cells) were seen outside of the graft (Figure 6D), these TH+ fibers adjancent to the transplant must have been of host origin. This latter finding suggests a non-cell autonomous or trophic effect of transplanting MEF2CA/R-NSCs on nearby host cells. Because the implanted grafts also contained uninfected cells, in addition to staining with anti-TH antibody, we co-stained with anti-GFP to identify the infected cells, and then counted the number of TH+/GFP+ DA neurons (Figure 6H). The infected MEF2CA/R-NSCs contained 15.1 ± 1.7% TH+ neurons (representing, in absolute numbers, 20,535 ± 3,630 TH+/mm^3^), whereas control-infected/R-NSCs contained only 1.9 ± 0.9% TH+ neurons (2,605 ± 1,116 TH+/mm^3^) (Figure 6I).

**Figure 7:**
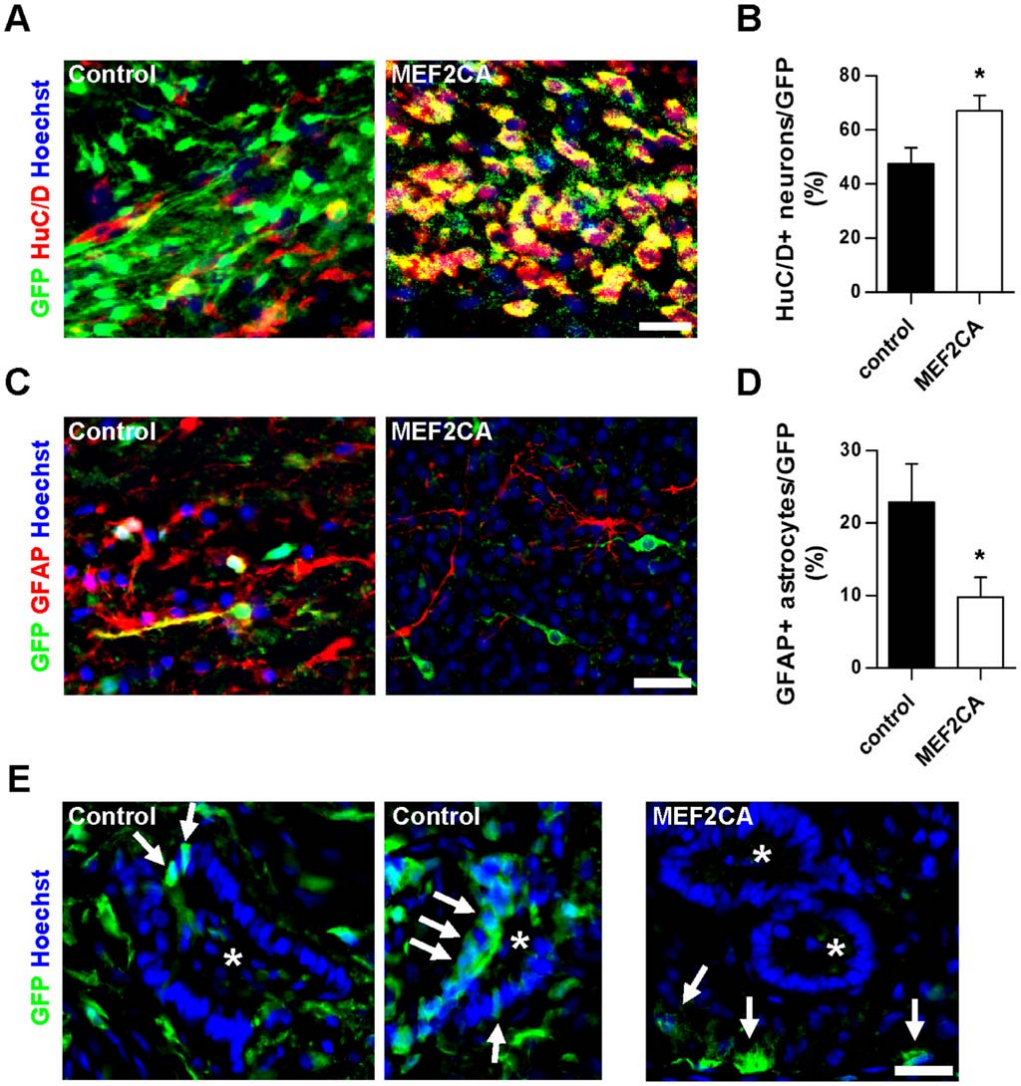
Neuronal differentiation of MEF2CA-infected R-NSCs in the striatum of 6-hydroxydopamine—lesioned rats. (A, C) Twelve weeks after transplantation, infected (GFP+) cells were analyzed with cell-type specific antibodies (anti-HuC/D for neurons and anti-GFAP for astrocytes, although neural progenitors can also be labeled with this marker). (B, D) Quantification of neuronal and astrocytic markers in control- and MEF2CA-infected cells. Ten random fields were selected in multiple sections at the same distance from the Bregma for each rat (*n* = 4). Values are mean + SEM, *n* = 10; *p<0.03 by *t*-test. (E) In transplanted brains, several regions manifested rosette structures (asterisks), consistent with hyperproliferation. For control-infected/R-NSC transplants (left-hand and middle panels), the GFP+ cells (arrows) were located within rosettes. MEF2CA/R-NSC transplants contained both MEF2CA-expressing cells (GFP+, green) as well as uninfected R-NSCs, but GFP+ cells (indicated by arrows) were located exclusively outside of the rosettes (right-hand panel). All scale bars: 25 µm.

We then further analyzed the cellular phenotypes of the engrafted/infected cells. We found that 47.4 ± 5.9% of control-infected/R-NSCs versus 67.0 ± 5.7% of MEF2CA/R-NSCs differentiated into human neuronal protein-positive (HuC/D+) neurons in the rat brain within twelve weeks of transplantation (Figure 7A and 7B). Additionally, 22.8 ± 5.2% versus 9.7 ± 2.7% of control- and MEF2CA-infected cells became GFAP+ astrocytes, respectively (Figure 7C, and 7D). These results indicate that MEF2CA expression was significantly better than control (*p*<0.03) in enhancing differentiation into neurons over astrocytes *in vivo*.

Most importantly, over a greater than six-month period we did not observe cellular hyperproliferation, hESC-derived teratomas, or Oct4/alkaline phosphatase-positive cells developing from MEF2CA/R-NSCs transplanted into the rat brain. Using anti-proliferating cell nuclear antigen (PCNA) staining as an index of proliferation, we observed <1% proliferation of these engrafted cells twelve weeks after transplantation (Figure S6C), less than that previously reported for growth factor-induced TH+ differentiation from hESCs at this time point [6], [7]. In contrast, twelve weeks after transplantation, control-infected R-NSCs frequently manifested features of hyperproliferation such as rosette formation. These changes were visualized as a radial arrangement of cells after staining with either Hoechst dye or hematoxylin/eosin, and these rosettes contained control-infected/GFP-positive cells (Figure 7E, Control; rosettes marked by asterisks). Although we also observed rosettes in brains injected with MEF2CA-infected/R-NSCs, MEF2CA-infected/GFP-positive cells were never found within the rosettes (Figure 7E, MEF2CA; asterisks). As stated above, the brains transplanted with MEF2CA-infected (GFP-positive)/R-NSCs also received a percentage of uninfected (GFP-negative)/R-NSCs; it was in fact these uninfected R-NSCs that formed the hyperproliferating rosettes in the grafts. These data indicate that R-NSCs not containing MEF2CA retained an undifferentiated phenotype after transplantation and underwent hyperproliferation, unlike R-NSCs expressing MEF2CA.

## Discussion

Parkinson's disease (PD) is currently treated with dopamine agonists, although fetal cell-based therapy has also been attempted [46]–[48]. Pharmacological agents treat symptoms but do not restore DA neurons in PD patients. In addition, long-term treatments with dopamine agonists such as l-DOPA cause dyskinesias and eventually become ineffective. To overcome these disadvantages, cell therapy using fetal mesencephalic brain tissue has been employed, but the results have been mixed and largely unsatisfactory. One reason for the failure of such transplants is graft-induced dyskinesias; in fact, many of these grafts contain more serotonin and GABAergic neurons than DA neurons. Both pulsatile delivery of DA agonists and upregulation of GABA_A_ receptors have been shown to contribute to dyskinesias in primates [49], so we reasoned that improved production of DA neurons might improve this situation. An additional problem has been extensive cell death in the grafts [46]. Therefore, strategies to enrich for DA neurons, especially of the A9 subtype rather than other neuronal types, and to prolong cell survival are likely to result in improved cell-based therapies for PD.

hESC-derived DA neuronal precursors have been considered as an alternative source for cell-based therapies in PD. Although this approach holds promise, hESC-based therapies face several hurdles, including death of engrafted cells, failure of migration/incorporation into host brain, lack of differentiation into appropriate neuronal cell types, and formation of tumors after transplantation. One technical advance that we propose here to overcome these problems is the forced expression of a transcription factor that drives more restricted neuronal lineage choices from ESCs, while also fostering survival of the differentiated cells. In this study, we present evidence that MEF2C represents a transcription factor that can accomplish these goals. Our feeder-free/neurosphere-based protocol for isolating rosettes followed by transduction with MEF2CA addresses many of the issues associated with the production of human stem cells for therapeutic use in the brain. This approach is particularly well suited for PD since, unlike previous methods [7], a substantial number of dopaminergic neurons can be generated in the absence of hyperproliferating cell types. Previously, we and others have shown that the MEF2 transcription factors, with MEF2C the predominant isoform during early brain development, represent a family of activity-dependent neurogenic effectors that enhance survival and neuronal differentiation [18]–[20], [50]. Moreover, we have recently shown that conditional knockout of *Mef2c* in mice during very early brain development – at the NPC stage – impairs neurogenesis, migration, and synaptogenesis *in vivo*, resulting in a behavioral phenotype resembling Autism-spectrum disorders [19].

Here, we initially investigated the function of MEF2C during early neurogenesis of hESCs *in vitro* by knocking down endogenous MEF2C using shRNAs at the R-NSC/NPC stage. At this stage, MEF2C expression was maximal, and cells homogenously expressed nestin and musashi1. As a result, we found a reduction in the number of hESC-derived neurons and dendritic/synaptic spines on those neurons, fitting well with our previous *in vivo* results in mice when we knocked out MEF2C at the NPC stage [19].

Conversely, when we overexpressed MEF2CA *in vivo*, we drove neurogenesis from hESC-derived R-NSCs/NPCs, specifically producing an enrichment of the dopaminergic phenotype. We found that MEF2C-mediated neurogenesis is not simply due to neuronal survival. For example, to distinguish neurogenic vs. survival effects of MEF2CA, we included Bcl-xL as a control and found that it did not significantly increase neuronal differentiation. Previously, both growth factors and transcription factors have been used to increase the percentage of NSCs that express neuronal and dopaminergic phenotypes [5], [51], but no method has proven to be totally effective. In this regard, a critical consideration is that if 100% of the hESCs do not become terminally differentiated as neuronal cells, then hyperproliferation can occur with possible tumorigenic potential. In contrast, with our approach, the great majority of cells receiving MEF2CA became neuronal, which can overcome this conundrum. Thus, our findings suggest that MEF2C is an effective driver of neurogenesis and in the proper context its expression prevents hyperproliferation. Additionally, our results show that under our conditions expression of MEF2C produces substantial enrichment in the dopaminergic phenotype. We show that the mechanism for this dopaminergic effect includes up-regulation by MEF2C of two additional transcription factors, nurr1 and the ETS family member Etv1. In the future, with more efficient transduction of MEF2CA or sorting of MEF2CA-positive cells, coupled with transient expression of MEF2CA only during the critical neurogenesis stage, this approach may provide an even more valuable source of human neural progenitor cells that are programmed to become dopaminergic neurons after transplantation in PD patients.

Our *in vivo* experiments using 6-OHDA-lesioned Parkinsonian rats and implanted lenti-MEF2CA/R-NSCs revealed good survivability for at least 6 months after transplantation, possibly because of the anti-apoptotic properties in addition to the neurogenic effect of MEF2CA [18], [29]. Most importantly, we observed significant neurobehavioral/motoric improvement after transplantation of MEF2CA/R-NSCs in this PD animal model compared to control/R-NSCs. By comparing control/R-NSCs vs. MEF2CA/R-NSCs in the same experimental model, we controlled for nonspecific transplantation-related effects on behavior. Prior studies had observed comparable improvement in motor function in the same Parkinsonian rat model after transplanting hESCs that had been co-cultured with telomerase-immortalized human mesencephalic astrocytes to enhance differentiation of the stem cells into dopaminergic neurons; however, this improvement was accompanied by hyperproliferation, heralding potential tumor formation [7]. Our approach using MEF2CA-transduced stem cells can avoid this major deterrent to transplantation.

In conclusion, our results demonstrate that MEF2C restricts hESCs to the neuronal lineage and that this attribute can be used to generate neurons and avoid tumor formation when used for cell-based therapies. Furthermore, hESC/NPCs programmed to become neurons via MEF2C activity were protected from apoptosis and enriched for the dopaminergic phenotype. This approach could potentially provide a limitless supply of stem cells for therapeutic application in PD. Our technique represents a unique approach for the production of cells for regenerative medicine, while at the same time avoiding apoptosis and tumorigenesis by promoting directed differentiation with a pro-survival/neurogenic transcription factor.

## Materials and Methods

### Experimental Animals

The Institute's Animal Care and Use Program is accredited by the AAALAC International and a Multiple Project Assurance A3053-1 is on file in the OLAW DHHS. Animal Usage Form 08–054, “Rat Model for Parkinson's Disease,” approval date April 16, 2008.

### hESC Culture and Neural Induction

Undifferentiated H9 hESCs (WiCell Research Institute) of passage 48–69 were cultured on a feeder layer of γ-irradiated human foreskin fibroblasts (Hs27, ATCC)/0.1% gelatin in growth medium (DMEM/F12, 20% knockout serum replacement, 1 mM non-essential amino acids, 0.1 mM β-mercaptoethanol (Invitrogen)) supplemented with 8 ng bFGF/ml (Sigma). Cells were subcultured once a week, and the medium changed everyday. For neural induction, hESCs were incubated in neural induction medium (NIM; DMEM/F12:Neurobasal (1∶1), 2% B27, 1% N2 (Invitrogen)) for 24 h and dissociated into small clumps by mechanical scraping. Small clumps were transferred to a bacterial Petri dish in NIM for an additional 3 days. The small clumps, termed neuroectodermal spheres (NES), were transferred to a new Petri dish for a 6-day incubation in neural proliferation medium (NPM; DMEM/F12:Neurobasal (1∶1), 1% B27, 0.5% N2, 20 ng bFGF/ml, and 20 ng EGF/ml (R&D)). These NES were replated onto laminin (LN, 10 µg/ml)-coated cell culture dishes in NPM for 2–3 days to form rosettes. Under a stereomicroscope (Leica), rosettes were isolated with a needle, transferred to a bacterial Petri dish and cultured in NPM for 3 days to 1 month. At this rosette stage, cells were designated rosette-neural stem cells (R-NSCs). These R-NSCs were dissociated into a single cell suspension with Accutase (Chemicon) and plated onto poly-l-ornithine (PLO, 10 µg/ml)/LN (1 µg/ml)-coated cell culture plates in NPM. At this stage, cells were designated neural progenitor cells (NPCs) and formed a monolayer. One week later, the cells were dissociated again and replated onto PLO (100 µg/ml)/LN (10 µg/ml) in terminal differentiation medium (TDM; DMEM/F12:Neurobasal (1∶1), 1% B27, 10 ng BDNF/ml (R&D), and 10 ng GDNF/ml (R&D)). During the first two weeks after this plating, the cells began to stain for immature neuronal markers and were therefore designated Neural Stage I. Cells differentiating for 15 to 28 days after plating were designated Neural Stage II, and greater than 28 days, Neural Stage III. During these stages, we examined the expression of neuronal, astrocytic and oligodendrocytic markers. Total RNA and cell lysates were collected from each representative stage for quantitative PCR and immunoblotting.

### Lentiviral Constructs and Cell Infection

Lentiviral transfer vectors were transfected into HEK293 cells to generate each lentivirus (see Text S1). For infection of hESC-derived cells, we used cells at the R-NSC stage. Infected R-NSCs were dissociated and plated onto PLO (10 µg/ml)/LN (1 µg/ml)-coated plates in NPM in order to grow hNPCs in monolayer culture for *in vitro* experiments. At confluence, approximately 14 days after infection, these hNPCs were dissociated, plated onto glass coverslips coated with PLO (100 µg/ml)/LN (10 µg/ml), and terminally differentiated in TDM. The differentiated cells were fixed at various stages for immunostaining. For the *in vivo* transplantation experiments, infected R-NSCs were allowed to differentiate for 7–10 days as nestin/musashi1-positive neurospheres, and then dissociated just prior to injection into the striatum.

### Quantitative RT- PCR

Total RNA (500 ng) obtained from cells at various time points were used to make cDNA. The expression level of each gene was normalized to endogenous GAPDH. Fold change in gene expression was calculated using the Pfaffl equation [52]. For detailed information concerning RNA isolation, reverse transcription, and qPCR, see Text S1.

### TUNEL Assay

R-NSCs were infected with lenti-scrambled control shRNA or lenti-shMEF2Cs and differentiated in TDM *in vitro*. Cells were fixed at 14 or 33 dpi, and apoptotic cells were labeled with the ApopTag *In Situ* Apoptosis Detection Kit (Chemicon; see Text S1).

### Immunocytochemistry/Immunohistochemistry

Cells in culture were fixed and permeabilized for staining. Parkinsonian rat brains were sectioned at 15 µm by cryostat, treated with Antigen unmasking solution (Vector), and permeabilized before staining with primary antibodies and fluorophore-conjugated secondary antibodies. Detailed information related to fixation, antibodies, dilution, and image analysis are listed under Text S1.

### *In Vitro* Electrophysiology

GFP-labeled NPCs were sorted by FACS to provide a pure population of lenti-infected cells for subsequent electrophysiological recording. The cells were differentiated in TDM for at least 5 weeks, and then analyzed for neuronal electrophysiological properties by patch-clamp recording (see Text S1).

### Chromatin Immunoprecipitation (ChIP) Assay

R-NSCs/NPCs were dissociated into single cells, cross-linked, lysed, and sonicated as described in the Text S1. The supernatant was used for immunoprecipitation with IgG or anti-MEF2 antibody. DNA fragments were purified and used for qPCR (see Text S1).

### 6-Hydroxydopamine (6-OHDA) Lesions, Transplantation, Immunosuppression, and Behavioral Tests

Sprague-Dawley rats with unilateral 6-OHDA lesions of the nigrostriatal pathway were monitored by apomorphine-induced rotations after transplantation with MEF2CA-programmed R-NSCs or control cells. Rats were housed and handled in accordance with the guidelines of Institutional Animal Care and Use Committee of Sanford-Burnham Medical Research Institute. For detailed information on behavioral tests and cell transplantation, see Text S1.

### Statistical Analysis

Data are reported as mean ± SEM. Statistical tests in each experiment are listed in the figure legends or text. All data were analyzed using the Prism 5 program **(**GraphPad Software, Inc.). Statistical significance between two experimental groups was assessed with a one-tailed Student's *t*-test. For analysis of data from three or more groups with a single independent factor that was variable, a one-way ANOVA with post hoc Tukey's multiple comparison test was used. A two-way ANOVA with planned comparisons was used for analysis of data among multiple pairs with two independent factors.

## Supporting Information

Text S1 **Supplemental methods and references.**
(PDF)

Figure S1 **Endogenous expression of stage-specific genes during neural differentiation monitored by qPCR.** (A, B) Total RNA was isolated from cells at the hESC, NES, R-NSC, NPC, Neural I, Neural II, and Neural III stages for quantitative RT-PCR. Note that the RNA levels of myelin basic protein (MBP, from oligodendrocytes) and S100β (from astrocytes) increased at Neural Stage III, at which time neuronal maturation was also occurring. Syn I, Synapsin I. Values are mean + SEM, *n* = 3; *p<0.05 compared to hESC by ANOVA. (C) Neurons from Neural Stage III stained with anti-PSD95 and -Synapsin I (Syn I) antibodies. Arrows indicate the clusters showing juxtaposition of PSD95 to Synapsin I. Scale bar: 5 µm.
(TIF)

Figure S2 **Validation of shRNAs against MEF2C.** (A) DNA constructs of the target reporter and shRNAs for MEF2C. (B) Scrambled or shRNAs directed against MEF2C (shMEF2C-1, -2, or -3) were co-transfected with a target reporter into HEK293 cells. Four days later, cells were harvested for immunoblot using anti-GFP (to detect the target reporter), anti-turbo GFP (for scrambled or shMEF2Cs), or anti-actin (as a loading control). An siRNA against MEF2C (siMEF2C) was used as a positive control. (C) hESC-derived R-NSCs were infected with scrambled, lenti-shMEF2C-1 or -2. Fluorescent images were taken at 20 days post infection (dpi).
(TIF)

Figure S3 **Construction of lenti-MEF2CA viral construct and scheme for the infection of hESC-derived R-NSCs.** (A) Diagram of the lentiviral transfer vector harboring PGK promoter-MEF2CA-IRES2-GFP. (B) GFP expression level was measured by immunoblot to monitor the efficacy of infection of SH-SY5Y cells with lenti-control, -Bcl-xL or -MEF2CA viruses at different multiplicities of infection (MOI). Actin served as a loading control. (C) Transcriptional activity of lenti-MEF2CA was measured using a MEF2 RE-MHC-luciferase reporter gene. Values are mean + SEM, *n* = 3; ***p<0.001 by ANOVA. (D) Scheme for infection of R-NSCs with lenti-MEF2CA or control constructs and analysis of resulting cells (numbers indicate days in culture; refer to Figure 1A and Materials and Methods for details). (E) Infection efficiency of R-NSCs by each lentiviral construct was calculated by counting the ratio of GFP+ to total DAPI+ cells. Values are mean + SEM, *n* = 9. MEF2CA, constitutively active MEF2C; IRES, internal ribosome entry site; MEF2 RE, MEF2 response element.
(TIF)

Figure S4 **Neurogenic effect of MEF2CA on human fetal brain-derived neural progenitor cells (hFB-NPCs).** (A) Schematic diagram showing the two differentiation procedures used here. (B) Fluorescent images of cells infected by lenti-control or -MEF2CA virus. For assessment of neuronal markers, hFB-NPCs were differentiated by the upper protocol shown in (A). Cells were double labeled with an anti-GFP to identify viral-infected cells and anti-TuJ1 to identify newly generated neurons, or with anti-GFAP to label neural precursor cells or astrocytes. Scale bar: 25 µm. (C) Quantification of fluorescent marker data after differentiation of control-infected and lenti-MEF2CA—infected cells. Plots show TuJ1+ and GFAP+ versus total cells (*left*), and TuJ1+ and GFAP+ versus GFP+/infected cells (*right*). Values are mean + SEM, *n* = 10; **p<0.01, ***p<0.001 by ANOVA. (D) The longest neuronal process per cell was measured with Neuron J software. Values are mean + SEM, *n* = 50 cells counted for each lentiviral infection; ***p<0.0001 compared to control by ANOVA.
(TIF)

Figure S5 **Enrichment of DA neuronal markers and promoter analysis of DA neuron-related genes in MEF2CA-infected cells derived from R-NSCs.** (A) Relative mRNA levels of GIRK2 and CD28k were assessed throughout development *in vitro*. Values are mean + SEM, *n* = 3; *p<0.001 for values greater than in Neural Stage I by ANOVA. (B) Endogenous expression of MEF2C and nurr1 in MEF2CA-infected cells were analyzed by qPCR during Neural Stage III at 40 dpi. Values are mean + SEM, *n* = 3; **p<0.003, ***p<0.0002 compared to respective control by *t*-test. (C) Schematic diagram of putative MEF2 binding sites in the enhancer/promoter of various DA neuron-related genes. (D) Effects of various MEF2C constructs on nurr1 promoter activity. HeLa cells were cotransfected with empty vector, dominant negative MEF2C (MEF2DN), full-length wild-type MEF2C, or constitutively active MEF2C (MEF2CA) plus a nurr1 promoter (1.3 kb)-luciferase construct. Values are mean + SEM, *n* = 3; ***p<0.001 by ANOVA. (E) ChIP analysis of MEF2C association with the nurr1 promoter. After chromatin immunoprecipitation with anti-MEF2 antibody, qPCR primers detected the MEF2C response element in the nurr1 promoter region. Values are mean + SEM, *n* = 3; **p<0.01 by *t*-test.
(TIF)

Figure S6 **Infection efficiency of control-lentiviral vector and lenti-MEF2CA in hESC-derived R-NSCs.** (A) Control- or MEF2CA-infected R-NSCs were grown as neurospheres for one week in preparation for their transplantation. Note that the MEF2CA construct bears an IRES, which results in weaker expression of GFP. (B) Infection efficiency of control-lentiviral vector and lenti-MEF2CA. To count infected cells, an aliquot of R-NSCs was plated onto poly-l-ornithine/laminin-coated coverslips and stained with anti-GFP antibody. Values are mean + SEM, *n* = 9. (C) Twelve weeks after transplantation, 0.9±0.15% of the engrafted MEF2CA/R-NSCs (green) expressed PCNA (red). Arrows indicate PCNA+ cells among transplanted cells; *n* = 13 experiments with 2,200 cells scored (quantified in histogram at *right*). Scale bar: 25 µm.
(TIF)

Figure S7 **Increase in tyrosine hydroxylase-positive neuropil in host tissue adjacent to graft.** Tissue sections were stained for tyrosine hydroxylase (TH), and the intensity of staining in the neuropil adjacent to the graft was measured. Random fields (*n* = 7 randomly chosen fields for each of 4 animals) were interrogated and adjusted for background staining by subtracting the intensity of a similar field distal to the graft. Values are mean + SEM, *p<0.0001 by *t*-test.
(TIF)
