# Supplementary material for: MEF2C Enhances Dopaminergic Neuron Differentiation of Human Embryonic Stem Cells in a Parkinsonian Rat Model
Source: PLoS One. 2011 Aug 25;6(8):e24027. doi: 10.1371/journal.pone.0024027 (PMC3162026; doi:10.1371/journal.pone.0024027)
Supplement: Text S1 — Supplemental methods and references. (PDF) [file pone.0024027.s001.pdf]

Supporting Information

MEF2C Enhances Dopaminergic Neuron Differentiation of Human Embryonic Stem Cells in a Parkinsonian Rat Model

Eun-Gyung Cho, Jeffrey D. Zaremba, Scott R. McKercher, Maria Talantova, Shichun Tu, Eliezer Masliah, Shing Fai Chan, Nobuki Nakanishi, Alexey Terskikh, Stuart A. Lipton

Supplemental Methods

Lentiviral Vectors and Promoter-Luciferase Constructs

The lentiviral transfer vector pRRL-**PGK** promoter-**GFP**-SIN18WPRE (**PGK-GFP**), packaging plasmid pCMVΔR8.74, and envelope plasmid pMD.G are described elsewhere [1,2]. The lentiviral transfer vector pRRL-**PGK** promoter-**IRES2**-**GFP**-SIN18WPRE (**PGK-IRES2-GFP**) was used for insertion of MEF2CA or Bcl-xL. MEF2CA was generated by direct fusion of the MADS/MEF2 domains (612 amino acids) of human MEF2C with the transcriptional activation domain of VP16 (78 amino acids). The fused construct was amplified with primers containing BamHI sites and ligated between the PGK promoter and IRES2 in the PGK-IRES2-GFP vector (PGK-**MEF2CA**-IRES2-GFP). Human Bcl-xL in pcDNA3 was provided by Dr. John C. Reed at the Sanford-Burnham Medical Research Institute. Myc-tagged Bcl-xL was amplified using primers with EcoRV sites and then ligated into the SmaI site between the PGK promoter and IRES2 in the PGK-IRES2-GFP vector (PGK-**Bcl-xL**-IRES2-GFP).

The nurr1 promoter (-1329/+132) in a β-galactosidase reporter was kindly provided by Dr. Evelyn Murphy at University College Dublin, Ireland. This promoter was inserted into the pGL3-promoter vector (Promega) to generate a nurr1 promoter-luciferase construct. The Etv1 enhancer/promoter (-2.1 kb from transcription initiation site) was amplified from the GenomeWalker library (BD) and ligated into the pGL3-promoter vector.

The target reporter (PGKp-GFP-hMEF2C-3'UTR in pDsRed2-N1) used for validation of shRNAs against hMEF2C was provided by Dr. Mark Mercola at the Sanford-Burnham Medical Research Institute.

Generation and Characterization of Lentiviral Constructs

Lentiviral transfer vectors included PGK-GFP (control), PGK-MEF2CA-IRES2-GFP (lenti-MEF2CA), PGK-Bcl-xL-IRES2-GFP (lenti-Bcl-xL), shRNAs against MEF2C generated in pGIPZ-shRNAmir (shMEF2C-1 and shMEF2C-2), and non-silencing shRNA in pGIPZ-shRNAmir (scrambled shRNA control). Lentiviral constructs were prepared in house, except for shRNAs, which were obtained from a commercial source (Open Biosystems). Each lentiviral transfer vector was transduced into HEK293 cells with pCMVΔR8.74 and pMD.G to generate a replication-deficient lentivirus. On day 0, HEK293T cells were plated in 150-mm dishes with 15 x 10^6 cells/dish. On day 1, cells (~80% confluent) were transfected with three plasmids (lentiviral transfer vector, pCMVΔR8.74, and pMD.G) mixed in a ratio of 3:2:1 (30, 20 and 10 μg, respectively) and brought up to 1350 μl with H_2O; 150 μl of 2.5 M $CaCl_2$ were added drop wise. Finally, 1500 μl of 2x Hanks' balanced salt solution (HBSS: 8 mg NaCl, 0.37 mg KCl, 0.19 mg $Na_2HPO_4 \cdot 7H_20$, 1 mg glucose, 5 mg HEPES, pH 7.2 in 500 ml total volume) were added drop wise with constant vortexing. The mixture was incubated for 15 min at room temperature and applied to the cells in 16 ml of medium. Cells were incubated for 7 h in a 5% CO_2 incubator at 37 °C. Then, the medium was replaced with viral harvesting medium (Ultraculture medium, Bio-Whittaker) and further incubated for 48 h. On day 3, the medium was collected and centrifuged for 10 min at 400 x g and filtered with 0.45 μm pore diameter, low protein binding-cellulose acetate. To concentrate virus, the filtrate was centrifuged at 20,000 rpm (Beckman SW32) for 2.5 h at 4 °C. The viral pellet was resuspended in 100-150 μl of supernatant for 3 h at 4 °C. The concentrated virus was stored in aliquots at -80 °C. For titration, HEK293T cells were plated in 12-well plates with 2 x 10^5 cells/well one day before infection. Cells were infected with various volumes of virus (0.1, 0.3, 1.0, and 3.0 μl) the following day and incubated for 6-7 h. After washing twice with PBS, cells were further incubated in fresh growth medium for 3 days. Cells were trypsinized and resuspended in 2% FBS/PBS, and GFP

positive cells quantified by FACSCanto. A viral transfection unit (TU/ml) was calculated as follows: ((%GFP/100) x cell number at day of infection)/virus volume.

To measure MEF2CA activity after transduction with a lenti-MEF2CA construct, SH-SY5Y cells were plated on 24-well plates at 1 x 10^5 cells/well. The next day, each viral construct was added at a MOI of 0.5 for 7 h, washed once with PBS, and cultured for an additional day. Cells were transfected using Fugene HD (Roche) with 0.5 μg of MEF2 response element (RE)-MHC-Luciferase plasmid and 0.05 μg of Renilla as an internal control in each well. Two days later, cells were washed, lysed with 1x Passive lysis buffer (Promega), and quantified using the Dual-Luciferase reporter assay system (Promega). To monitor the expression of GFP after infection, SH-SY5Y cells infected with a MOI of 0.5 or 1 were lysed with 1x SDS buffer without reducing agent or bromophenol blue and quantified by BCA (Pierce). Rabbit anti-GFP antibody was used for probing GFP expression.

Infection of hESC-Derived R-NSCs with Lentiviral Constructs

R-NSCs were treated with Accutase (Chemicon) to make a single-cell suspension and transferred onto low-attachment plates (24-well plate, Costar) containing 300 μl NPM/well. Each lentiviral construct was added and incubated for 5 h, at which point cells were transferred without washing onto a 6-well low-attachment plate with 1.5 ml NPM/well. The next day, infected cells were washed once with PBS and placed back onto low-attachment plates in NPM for one week to allow for expansion. Infected cells were dissociated and plated onto PLO (10 μg/ml)/LN (1 μg/ml)-coated plates in NPM, at which time they had become hNPCs in monolayer culture. Confluent hNPCs were dissociated and replated in TDM on glass coverslips coated with PLO/LN for terminal differentiation. To assess changes over time in culture, these cells were stained with anti-GFP (to identify cells infected with control lentivirus, lenti-MEF2CA or lenti-

Bcl-xL) or with anti-turbo GFP (to identify cells infected with lenti-scrambled shRNA or lenti-shMEF2Cs), and anti-doublecortin or anti-MAP2 (to identify neurons).

Lentiviral Infection and Differentiation of Human Fetal Brain-Derived Neural Progenitor Cells (hFB-NPCs)

hFB-NPCs were dissociated with Accutase and infected with control (PGK-GFP, 1 x 10^9 IU/ml) or lenti-MEF2CA (PGK-MEF2CA-IRES2-GFP, 1 x 10^8 IU/ml) for 6 h. Infected cells were washed twice with PBS and placed into proliferation medium (Neurobasal medium/2% B27 supplemented with 20 ng bFGF/ml and 20 ng EGF/ml) for 5 days in order to form neurospheres. The neurospheres were then plated onto coverslips coated with PLO (50 μg/ml)/LN (5 μg/ml) in differentiation medium (bFGF/EGF-free neurobasal/2% B27 medium) for 3 days prior to assessment of neuronal marker expression. Alternatively, infected cells were directly plated onto PLO/LN-coated coverslips, incubated for one day in proliferation medium, and then cultured for 7 days in differentiation medium prior to measurement of neurite length; this alternative plating technique was used in order to obtain a monolayer of cells for neurite assessment. The longest neurite was picked and the length was measured by NeuronJ (an ImageJ plug-in) [3].

Enhancer/Promoter Analysis

Promoter regions, including the transcriptional start site (+1) of each gene, were retrieved using the transcriptional regulatory element database (TRED); MEF2 binding sites were predicted by PROMO using v. 8.3 of TRANSFAC software with a 10% maximum matrix dissimilarity rate.

Quantitative RT-PCR and Immunoblot Analyses

Endogenous expression of each gene of interest was assessed at different time points by qPCR using cDNA (miScript reverse transcription kit, Qiagen) obtained from total RNA (miRNAeasy mini kit, Qiagen). For qPCR, we used a LightCycler 480DNA SYBR green I master mix (Roche Applied Science) with the QuantiTect primer assay (Qiagen). The primers for endogenous MEF2C were chosen outside of the DNA-binding and MEF2 domains present in MEF2CA.

For immunoblots, cells from various stages of differentiation were harvested and lysed in cell lysis buffer (Cell signaling). Protein concentration was measured with BCA reagents (Pierce). Antibodies against MEF2C (1:1000, Aviva Systems Biology), MEF2D (1:1000, R&D), MEF2A (1:1000, Cell signaling), PSD95 (1:1000, Upstate), MAP2 (1:1000, Chemicon), Tau (1:1000, Santa Cruz), GFP (1:1000, Invitrogen), and actin (1:200,000, Chemicon) were used for specific protein identification. Horseradish peroxidase-conjugated anti-mouse or -rabbit IgGs (1:5000, Jackson ImmunoResearch) were used as secondary antibodies, and bands were developed with the ECL Plus Western blot detection system (GE Healthcare).

TUNEL Assay

Differentiated hNPCs infected with lenti-scrambled shRNA control or lenti-shMEF2Cs were assayed for apoptosis with the ApopTag *In Situ* Apoptosis Detection Kit (Chemicon) according to manufacturer's instructions with minor modifications. Precooled acetone:methanol (1:1) rather than ethanol:acetic acid (2:1) was used to post-fix cells. To identify cells infected with each virus, anti-turbo GFP antibody was added before mounting samples. Images were viewed by deconvolution microscopy using standard FITC (for turbo GFP signal), Cy3 (for signal labeled with ApopTaq), or DAPI excitation and emission filters.

Immunocytochemistry/Immunohistochemistry

Cells in culture were fixed with 4% paraformaldehyde for 10 min at 25 °C and then permeabilized with 0.25% Triton X-100 in PBS for 10 min. Parkinsonian rats were perfused with 4% paraformaldehyde, and, after removal from the cranium, brains were post-fixed for 4 h at 4 °C, then cryoprotected in 30% sucrose prior to freezing. Cryostat sections were cut at a thickness of 15 μm. Sections were soaked in Antigen Unmasking Solution (Vector) and microwaved for 30 s, followed by permeabilization with 0.25% Triton X-100 in PBS for 15 min. Primary antibodies were incubated for 16 h at 4 °C and fluorescence-conjugated secondary antibodies for 2 h at 25 °C. Numerous unstained cells in each field served as an internal control for staining specificity. Primary antibodies included: GFP (rabbit, 1:1000, Invitrogen), TuJ1 (mouse, 1:1000, Covance), MAP2 (mouse, 1:1000, Sigma), GFAP (mouse, 1:1000, Sigma), OCT4 (rabbit, 1:1000, Santa Cruz), vimentin (mouse, 1:1000, Sigma), Pax6 (mouse, 1:1000, Developmental Studies Hybridoma Bank (DSHB)), Sox2 (mouse, 1:1000, Chemicon), Musashi1 (Msi1, rabbit, 1:1000, Chemicon), doublecortin (DCX, guinea pig, 1:1000, Chemicon), Sl00β (rabbit, 1:10000, Swant), NeuN (mouse, 1:500, Chemicon), synaptophysin (Syn, rabbit, 1:1000, Dako), PSD95 (mouse, 1:500, Upstate), engrailed 1 (ENl, mouse, 1:100, DSHB), tyrosine hydroxylase (TH, mouse, 1:1000, Pel-Freez; rabbit, 1:1000, Chemicon), dopamine transporter (DAT, MAB369MI, 1:500, Millipore), HuC/D (mouse, 1:1000, Invitrogen), BrdU (mouse, 1:500, Chemicon). Alexa 488, 555 or 647 goat anti-mouse or -rabbit IgG (1:1000, Invitrogen) were used as secondary antibodies. Rhodamine Red conjugated Streptavidin (Pierce) was used for Biotin labeled anti-guinea pig or -mouse IgG. Images were generated by deconvolution microscopy; filters for DAPI, CY3, CY5, and FITC were used for three or four-color image capture, and a 'nearest neighbor' analysis was employed in the quantitative deconvolution software algorithm (Slidebook, Intelligent Imaging Innovations). For photomicrographs of grafts and host neuropil in the brains of parkinsonian rats, sections were incubated with anti-tyrosine hydroxylase antibody followed

by biotinylated secondary antibody (Vector) and avidin-conjugated peroxidase (Vector). Staining was visualized by incubating in diaminobenzidine (DAB) solution according to the manufacture's directions (Vector). Sections were scanned at a magnification of 20x (resolution of 0.5 μm/pixel (50,000 pix/inch)) using an Aperio ScanScope XT Automated High Throughput Scanner (Aperio Technologies). Background illumination levels were calibrated using a prescan procedure. For neuropil quantification, sections were imaged with a 60X objective using a bright field Olympus digital microscope. Parameters for images were: dimensions = 512 x 512 pixels; zoom = 3; gain = 40 – 45; offset = 15 – 20. DAB intensity associated with TH-immunoreactive structures was measured using NIH ImageJ software. Areas distal to the graft were imaged to provide intensity values as controls to subtract staining background for each tissue section.

Electrophysiology

To prepare cells for electrophysiological recording, R-NSCs were infected with a lenti-MEF2CA construct and kept on low-attachment plates for two weeks for cell expansion. R-NSCs were dissociated with Accutase into single cells that were then sorted into two groups (GFP+ and GFP-) using a FACSVantageSE DiVa (BD Biosciences). Sorted GFP+ cells were plated back onto low attachment plates and grown as neurospheres for two additional weeks in NPM in order to recover and expand, and then plated onto PLO (10 μg/ml)/LN (1 μg/ml)-coated dishes to become NPCs in monolayer. The GFP+/NPCs were plated onto 12-mm diameter glass coverslips coated with PLO (100 μg/ml)/LN (10 μg/ml) in 24-well plates at 5 x 10^4 cells/well in TDM. Five weeks later, whole-cell recordings were performed at room temperature. Briefly, coverslips were placed in a recording chamber with a volume of ~250 μl that was mounted on the stage of an Olympus IX71 inverted microscope. Electrical signals obtained with patch electrodes were amplified using an Axopatch 200B amplifier (Axon Instruments) and filtered at 5 kHz via a

Bessel low pass filter. Data were sampled and analyzed using pClamp 10.1 software (Molecular Devices). Patch pipettes were pulled from standard wall glass of 1.5 mm outer diameter (Warner) with final tip resistance of 5-12 MΩ. For recording voltage-gated Na^+ currents, the following intracellular solution was used (in mM): 120 CsCl, 20 tetraethylammonium chloride (TEA-Cl), 10 HEPES, 2.25 EGTA, 1 $CaCl_2$, 2 $MgCl_2$; pH adjusted to 7.4 with CsOH. To elicit voltage-gated currents, 100 ms depolarizing steps from -60 to +80 mV in 20 mV increments following a 300 ms prepulse to -90 mV were used. For recording ligand-gated currents, the intracellular solution contained (in mM): 130 Cs-gluconate; 2 MgATP, 1 $MgCl_2$; 10 EGTA; 10 HEPES; pH adjusted to 7.25 with CsOH. Osmolarity was adjusted to 300 mOsm with sucrose. The bath solution contained a saline based upon HBSS (in mM): 137 NaCl, 1 $NaHCO_3$, 0.34 Na_2HPO_4, 2.5 KCl, 0.44 KH_2PO_4, 2.5 $CaCl_2$, 5 HEPES, 22.2 glucose; pH adjusted to 7.3 with NaOH. Receptor agonists and antagonists were prepared in bath solution and applied by an array of tubes placed 75-100 μm from the cells. Drug administration was controlled by a series of rapidly-triggered valves (The Lee Company and Warner Instruments, USA). Solution changes were achieved within 50-100 ms, and a pipette containing bath solution was used to rapidly washout applied drugs.

Chromatin Immunoprecipitation (ChIP) Assay

ChIP assays were conducted as previously described with some modifications [4]. Briefly, about 20-30 million R-NSCs were used for each ChIP experiment. R-NSCs were dissociated with Accutase and crosslinked with 1% paraformaldehyde in PBS for 10 min at room temperature. Crosslinking was quenched by adding 125 mM glycine for 5 min. After washing with ice-cold PBS containing complete protease inhibitor cocktail (Roche Diagnostics), cells were pelleted and lysed in cell lysis buffer (1 mM EDTA, 10 mM HEPES/KOH, pH 7.9, 85 mM KCl, 0.5% NP40

and protease inhibitor cocktail) for 10 min at 4 °C. Nuclei were then pelleted by centrifugation at 3000 rpm for 10 min at 4 °C. The isolated nuclei were resuspended in nuclear lysis buffer (10 mM EDTA, 50 mM Tris-HCl, pH 8, 1% SDS, 0.5 % Empigen BB (Sigma) and protease inhibitor cocktail) and sonicated 10 times for 15 sec at maximum settings using a Misonix Sonicator (Misonix, Farmingdale, NY). Insoluble materials were removed by centrifugation at 14,000 rpm for 10 min at 4 °C. The supernatant was transferred to a new tube and diluted 10 fold in immunoprecipitation buffer (2 mM EDTA, 20 mM Tris-HCl, pH 8, 150 mM NaCl, 1% Triton X-100, and protease inhibitor cocktail). The lysates were pre-cleared with protein A-agarose (Sigma) for 2 hr at 4 °C, and 5% of each lysate saved as the input material. The rest of the lysate was used for immunoprecipitation by 10 μg of antibody (IgG or anti-MEF2 (C-21 or B-4); Santa Cruz Biotechnology). After overnight incubation at 4 °C, 50 μl of protein A-agarose (Sigma) was added to each reaction and incubated for 2 hr at 4 °C. The beads bound by immune-complexes were washed three times with each of the following buffers: low salt buffer (0.1 % SDS, 1 % Triton X-100, 2 mM EDTA, 20 mM Tris-HCl, pH 8, 150 mM NaCl) and TE buffer (10 mM Tris-HCl, pH 8, 1 mM EDTA). The immunoprecipitates were then eluted from the beads (elution buffer: 10 mM Tris-HCl, pH 8.0, 1 mM EDTA, pH 8.0, 1 % SDS) and de-crosslinked by incubating overnight at 65 °C. DNA was purified using QIAquick columns (Qiagen), following the manufacturer's instructions.

For qPCR, primers were designed to surround a region 150-250 bp from the putative MEF2 response elements in the Etv1 or nurr1 promoter, and used to determine the enrichment of specific sequences after ChIP. This enrichment was determined by qPCR using a SYBR Green-detection kit (Invitrogen) on a Mx3000P real time PCR system (Stratagene). Levels of enrichment after ChIP were calculated using the comparative cycle threshold method (Invitrogen) after normalizing with the IgG control. Primer sequences used for qPCR were as

follows: for the putative MEF2C response element in Etv1 promoter, 5'-cttgttcggatttttgctactacc-3' (forward), 5'-catagctaattaccctccgacagt-3' (reverse); for the putative MEF2 response element in nurr1 promoter, 5'-tgtgcttgagtttccgactg-3' (forward), 5'-cagcaactccagctcagagg-3' (reverse).

6-Hydroxydopamine Lesions, Transplantation, Immunosuppression, and Behavioral Tests

Adult male Sprague-Dawley rats (~8 weeks old, 224-332g) were injected on the left side of the medial forebrain bundle with 6-hydroxydopamine (6-OHDA) to produce unilateral lesions of the nigrostriatal pathway (surgery model #SU048, Taconic). One month after injection, as a baseline to evaluate the extent of 6-OHDA-induced lesion, apomorphine-induced rotations were measured following a standard protocol (Rotometer Photobeam Activity System, San Diego Instruments). Briefly, apomorphine (R-(-)-Apomorphine hydrochloride hemihydrate, 0.1 mg/ml in 0.9% NaCl, Sigma) was injected subcutaneously at a volume of 0.5 ml/kg body weight. Immediately after injection, the animal was placed into the rotometer and the time until the first full rotation recorded. The number of turns was recorded in 5-minute intervals for 30 minutes. Rats that had substantial deficits (>180 rotations/30 min) were divided randomly into two groups, one to receive control/R-NSCs and the other MEF2CA/R-NSCs. For stem cell transplantation, we followed previously published protocols [5,6]. Briefly, rats were transplanted with ~ 5-6 x 10^5 stem cells in 3 μl HBSS injected into the striatum, ipsilateral to the lesion (Coordinates from bregma: AP: 0, ML + 2.8 mm, at three different depths DV -6, -5, -4, as measured stereotactically). For transplantation, cells were dissociated with accutase into a single-cell suspension. Aggregates or clumps were removed by passage through a 35-μm mesh cell strainer. Dissociated cells were quantified microscopically and viability assessed by exclusion of 0.4% trypan blue. Viability was ~90%, and the transplanted cell number was based on the live-cell count. Rats were immunosuppressed with daily intraperitoneal (i.p.) injections of 20 mg

Cyclosporin A/kg (LC Laboratories) for 5 days, starting 2 days before transplantation. Thereafter, daily i.p. injections of Cyclosporin A at 15 mg/kg were given until sacrifice. Apomorphine test regimens were performed every two weeks after transplantation, following the procedures described above. Additionally, we assessed the effect of stem cell transplantation with the limb-use asymmetry (cylinder) test [7]. For this purpose rats were placed in a transparent cylinder (20 cm in diameter, 30 cm in height), and exploratory activity was recorded with a digital video camera for 10 minutes. Forelimb used in each of three behaviors was counted: initially contacting the wall of the cylinder when rearing, movements along the wall while rearing, and landing after rearing. As described previously, in each trial rats had to exhibit at least five such movements to be scored. Counts for initial wall contact and movements along the wall were combined for analysis. Asymmetry scores were calculated by the formula:

$$\frac{(\%M_n - \%M_i)_{wall} + (\%M_n - \%M_i)_{land}}{2} * 100$$

(%Mn = percent ipsilateral forelimb movements, %Mi = percent contralateral forelimb movements) [7].

Quantification of DA Neurons

Lenti-MEF2CA expressing hESCs were differentiated and plated under terminal differentiation conditions. Cells were fixed at Neural Stage I for rabbit anti-GFP and mouse anti-TH antibody staining, or at Neural Stage II for rabbit anti-GFP and mouse anti-EN antibodies staining. Two independent experiments were carried and five different fields from each experiment were quantified. Over 1200 cells were counted for TH+ neurons and over 500 cells were for EN+ cells.

For quantification of TH+ neurons from PD rats, brains were prepared and sectioned as described above (see **Immunocytochemistry/Immunohistochemistry** section**)**. Three random animals were chosen from both the control and MEF2CA groups, and ten random fields were

selected in multiple sections at the same distance from the Bregma for each rat. Each section was co-stained with rabbit anti-GFP and mouse anti-TH antibodies, and visualized with Alexa 488 goat anti-rabbit and Alexa 555 goat anti-mouse secondary antibodies. Images were taken under deconvolution microscopy and a 'nearest neighbor' analysis was employed as the deconvolution software algorithm (Slidebook, Intelligent Imaging Innovations).

References

1. Zufferey R, Dull T, Mandel RJ, Bukovsky A, Quiroz D, et al. (1998) Self-inactivating lentivirus vector for safe and efficient in vivo gene delivery. J Virol 72: 9873-9880.
2. Dull T, Zufferey R, Kelly M, Mandel RJ, Nguyen M, et al. (1998) A third-generation lentivirus vector with a conditional packaging system. J Virol 72: 8463-8471.
3. Meijering E, Jacob M, Sarria JC, Steiner P, Hirling H, et al. (2004) Design and validation of a tool for neurite tracing and analysis in fluorescence microscopy images. Cytometry A 58: 167-176.
4. Metivier R, Penot G, Hubner MR, Reid G, Brand H, et al. (2003) Estrogen receptor-alpha directs ordered, cyclical, and combinatorial recruitment of cofactors on a natural target promoter. Cell 115: 751-763.
5. Roy NS, Cleren C, Singh SK, Yang L, Beal MF, et al. (2006) Functional engraftment of human ES cell-derived dopaminergic neurons enriched by coculture with telomerase-immortalized midbrain astrocytes. Nat Med 12: 1259-1268.
6. Li Z, McKercher SR, Cui J, Nie Z, Soussou W, et al. (2008) Myocyte enhancer factor 2C as a neurogenic and antiapoptotic transcription factor in murine embryonic stem cells. J Neurosci 28: 6557-6568.

7. Schallert T, Fleming SM, Leasure JL, Tillerson JL, Bland ST (2000) CNS plasticity and assessment of forelimb sensorimotor outcome in unilateral rat models of stroke, cortical ablation, parkinsonism and spinal cord injury. Neuropharmacology 39: 777-787.

Figure S1 Endogenous expression of stage-specific genes during neural differentiation monitored by qPCR. (A, B) Total RNA was isolated from cells at the hESC, NES, R-NSC, NPC, Neural I, Neural II, and Neural III stages for quantitative RT-PCR. Note that the RNA levels of myelin basic protein (MBP, from oligodendrocytes) and S100β (from astrocytes) increased at Neural Stage III, at which time neuronal maturation was also occurring. Syn I, Synapsin I. Values are mean + SEM, $n = 3$; $*p < 0.05$ compared to hESC by ANOVA. (C) Neurons from Neural Stage III stained with anti-PSD95 and -Synapsin I (Syn I) antibodies. Arrows indicate the clusters showing juxtaposition of PSD95 to Synapsin I. Scale bar: 5 μm.

Figure S2 Validation of shRNAs against MEF2C. (A) DNA constructs of the target reporter and shRNAs for MEF2C. (B) Scrambled or shRNAs directed against MEF2C (shMEF2C-1, -2, or -3) were co-transfected with a target reporter into HEK293 cells. Four days later, cells were harvested for immunoblot using anti-GFP (to detect the target reporter), anti-turbo GFP (for scrambled or shMEF2Cs), or anti-actin (as a loading control). An siRNA against MEF2C (siMEF2C) was used as a positive control. (C) hESC-derived R-NSCs were infected with scrambled, lenti-shMEF2C-1 or -2. Fluorescent images were taken at 20 days post infection (dpi).

Figure S3 Construction of lenti-MEF2CA viral construct and scheme for the infection of hESC-derived R-NSCs. (A) Diagram of the lentiviral transfer vector harboring PGK promoter-MEF2CA-IRES2-GFP. (B) GFP expression level was measured by immunoblot to monitor the efficacy of infection of SH-SY5Y cells with lenti-control, -Bcl-xL or -MEF2CA viruses at different multiplicities of infection (MOI). Actin served as a loading control. (C) Transcriptional activity of lenti-MEF2CA was measured using a MEF2 RE-MHC-luciferase reporter gene. Values are mean + SEM, $n = 3$; $***p < 0.001$ by ANOVA. (D) Scheme for infection of R-NSCs

with lenti-MEF2CA or control constructs and analysis of resulting cells (numbers indicate days in culture; refer to Figure 1A and Materials and Methods for details). (E) Infection efficiency of R-NSCs by each lentiviral construct was calculated by counting the ratio of GFP+ to total DAPI+ cells. Values are mean + SEM, $n = 9$. MEF2CA, constitutively active MEF2C; IRES, internal ribosome entry site; MEF2 RE, MEF2 response element.

Figure S4 Neurogenic effect of MEF2CA on human fetal brain-derived neural progenitor cells (hFB-NPCs). (A) Schematic diagram showing the two differentiation procedures used here. (B) Fluorescent images of cells infected by lenti-control or -MEF2CA virus. For assessment of neuronal markers, hFB-NPCs were differentiated by the upper protocol shown in (A). Cells were double labeled with an anti-GFP to identify viral-infected cells and anti-TuJ1 to identify newly generated neurons, or with anti-GFAP to label neural precursor cells or astrocytes. Scale bar: 25 μm. (C) Quantification of fluorescent marker data after differentiation of control-infected and lenti-MEF2CA—infected cells. Plots show TuJ1+ and GFAP+ versus total cells (*left*), and TuJ1+ and GFAP+ versus GFP+/infected cells (*right*). Values are mean + SEM, $n = 10$; $^{**}p < 0.01$, $^{***}p < 0.001$ by ANOVA. (D) The longest neuronal process per cell was measured with Neuron J software. Values are mean + SEM, $n = 50$ cells counted for each lentiviral infection; $^{***}p < 0.0001$ compared to control by ANOVA.

Figure S5 Enrichment of DA neuronal markers and promoter analysis of DA neuron-related genes in MEF2CA-infected cells derived from R-NSCs. (A) Relative mRNA levels of GIRK2 and CD28k were assessed throughout development *in vitro*. Values are mean + SEM, $n = 3$; $^{*}p < 0.001$ for values greater than in Neural Stage I by ANOVA. (B) Endogenous expression of MEF2C and nurr1 in MEF2CA-infected cells were analyzed by qPCR during Neural Stage III

at 40 dpi. Values are mean + SEM, $n = 3$; $^{**}p < 0.003$, $^{***}p < 0.0002$ compared to respective control by *t*-test. (C) Schematic diagram of putative MEF2 binding sites in the enhancer/promoter of various DA neuron-related genes. (D) Effects of various MEF2C constructs on nurr1 promoter activity. HeLa cells were cotransfected with empty vector, dominant negative MEF2C (MEF2DN), full-length wild-type MEF2C, or constitutively active MEF2C (MEF2CA) plus a nurr1 promoter (1.3 kb)-luciferase construct. Values are mean + SEM, $n = 3$; $^{***}p < 0.001$ by ANOVA. (E) ChIP analysis of MEF2C association with the nurr1 promoter. After chromatin immunoprecipitation with anti-MEF2 antibody, qPCR primers detected the MEF2C response element in the nurr1 promoter region. Values are mean + SEM, $n = 3$; $^{**}p < 0.01$ by *t*-test.

Figure S6 Infection efficiency of control-lentiviral vector and lenti-MEF2CA in hESC-derived R-NSCs. (A) Control- or MEF2CA-infected R-NSCs were grown as neurospheres for one week in preparation for their transplantation. Note that the MEF2CA construct bears an IRES, which results in weaker expression of GFP. (B) Infection efficiency of control-lentiviral vector and lenti-MEF2CA. To count infected cells, an aliquot of R-NSCs was plated onto poly-L-ornithine/laminin-coated coverslips and stained with anti-GFP antibody. Values are mean + SEM, $n = 9$. (C) Twelve weeks after transplantation, 0.9 ± 0.15% of the engrafted MEF2CA/R-NSCs (green) expressed PCNA (red). Arrows indicate PCNA+ cells among transplanted cells; $n = 13$ experiments with 2,200 cells scored (quantified in histogram at *right*). Scale bar: 25 µm.

Figure S7 Increase in tyrosine hydroxylase-positive neuropil in host tissue adjacent to graft. Tissue sections were stained for tyrosine hydroxylase (TH), and the intensity of staining in the neuropil adjacent to the graft was measured. Random fields ($n = 7$ randomly chosen fields

for each of 4 animals) were interrogated and adjusted for background staining by subtracting the intensity of a similar field distal to the graft. Values are mean + SEM, $*p < 0.0001$ by *t*-test.
