# Supplementary figures and images for: MEF2C Enhances Dopaminergic Neuron Differentiation of Human Embryonic Stem Cells in a Parkinsonian Rat Model
Source: PLoS One. 2011 Aug 25;6(8):e24027. doi: 10.1371/journal.pone.0024027 (PMC3162026; doi:10.1371/journal.pone.0024027)

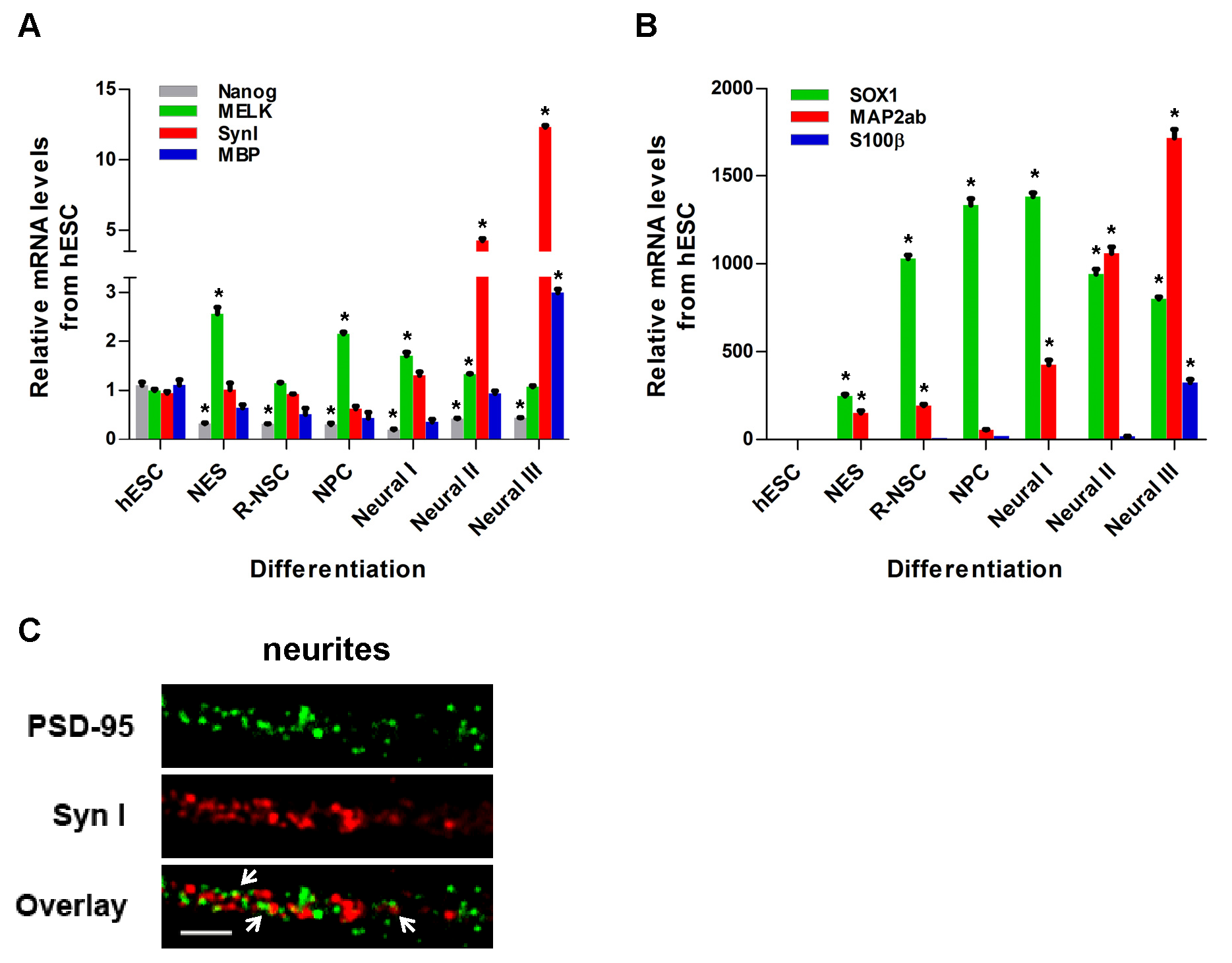

Supplement: Figure S1 — Endogenous expression of stage-specific genes during neural differentiation monitored by qPCR. (A, B) Total RNA was isolated from cells at the hESC, NES, R-NSC, NPC, Neural I, Neural II, and Neural III stages for quantitative RT-PCR. Note that the RNA levels of myelin basic protein (MBP, from oligodendrocytes) and S100β (from astrocytes) increased at Neural Stage III, at which time neuronal maturation was also occurring. Syn I, Synapsin I. Values are mean + SEM, n = 3; *p<0.05 compared to hESC by ANOVA. (C) Neurons from Neural Stage III stained with anti-PSD95 and -Synapsin I (Syn I) antibodies. Arrows indicate the clusters showing juxtaposition of PSD95 to Synapsin I. Scale bar: 5 µm. (TIF) [file pone.0024027.s002.tif]

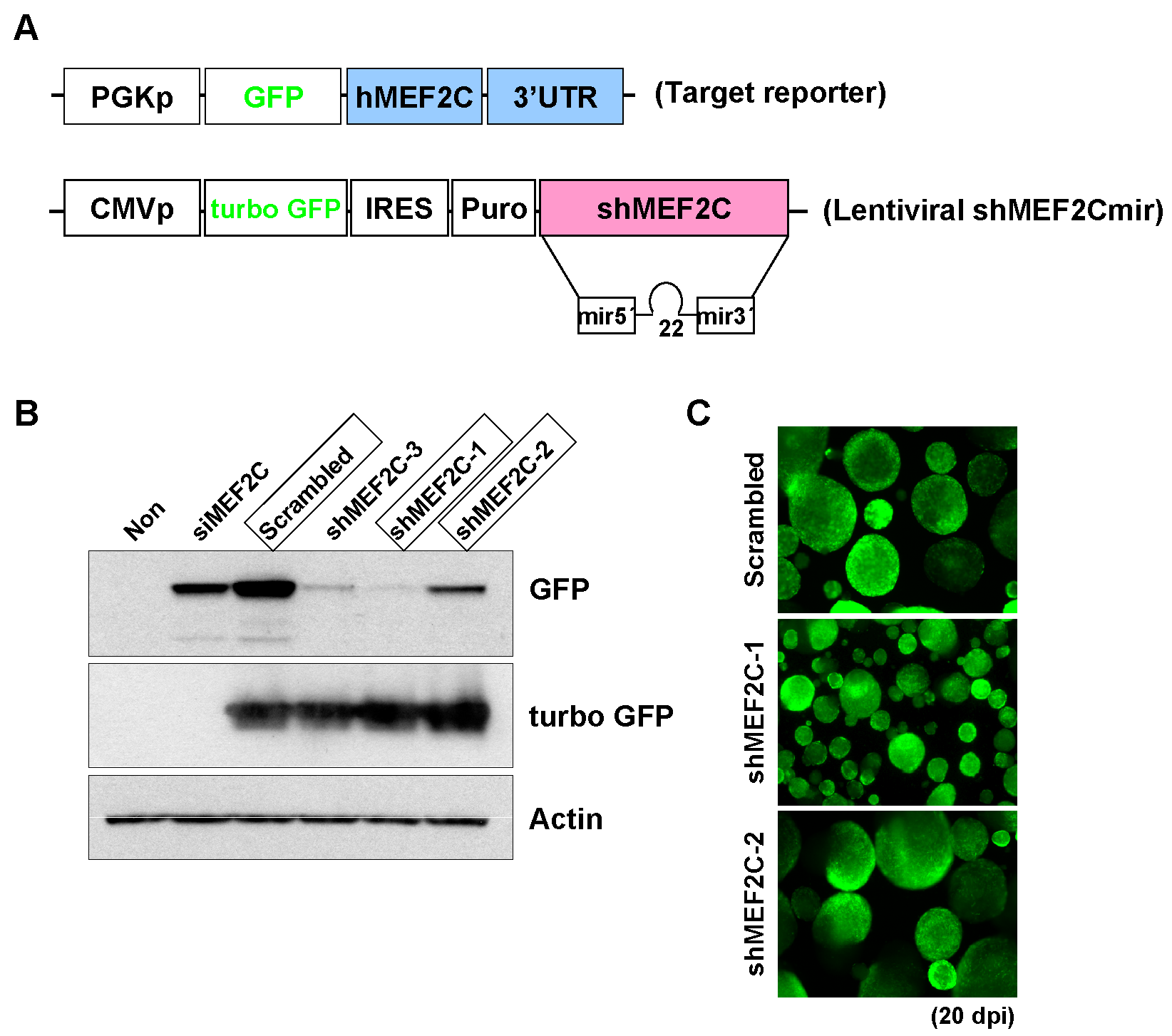

Supplement: Figure S2 — Validation of shRNAs against MEF2C. (A) DNA constructs of the target reporter and shRNAs for MEF2C. (B) Scrambled or shRNAs directed against MEF2C (shMEF2C-1, -2, or -3) were co-transfected with a target reporter into HEK293 cells. Four days later, cells were harvested for immunoblot using anti-GFP (to detect the target reporter), anti-turbo GFP (for scrambled or shMEF2Cs), or anti-actin (as a loading control). An siRNA against MEF2C (siMEF2C) was used as a positive control. (C) hESC-derived R-NSCs were infected with scrambled, lenti-shMEF2C-1 or -2. Fluorescent images were taken at 20 days post infection (dpi). (TIF) [file pone.0024027.s003.tif]

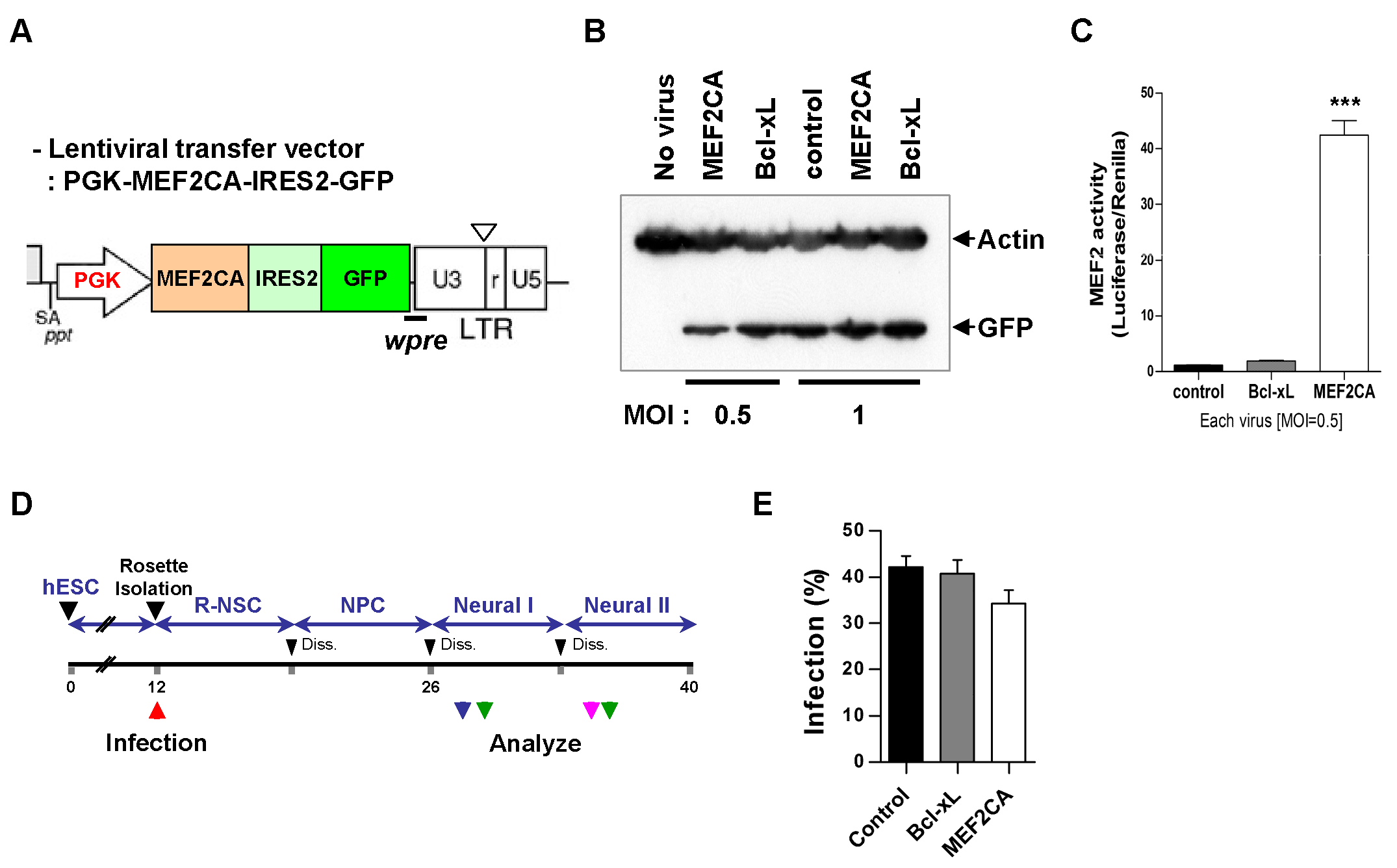

Supplement: Figure S3 — Construction of lenti-MEF2CA viral construct and scheme for the infection of hESC-derived R-NSCs. (A) Diagram of the lentiviral transfer vector harboring PGK promoter-MEF2CA-IRES2-GFP. (B) GFP expression level was measured by immunoblot to monitor the efficacy of infection of SH-SY5Y cells with lenti-control, -Bcl-xL or -MEF2CA viruses at different multiplicities of infection (MOI). Actin served as a loading control. (C) Transcriptional activity of lenti-MEF2CA was measured using a MEF2 RE-MHC-luciferase reporter gene. Values are mean + SEM, n = 3; ***p<0.001 by ANOVA. (D) Scheme for infection of R-NSCs with lenti-MEF2CA or control constructs and analysis of resulting cells (numbers indicate days in culture; refer to Figure 1A and Materials and Methods for details). (E) Infection efficiency of R-NSCs by each lentiviral construct was calculated by counting the ratio of GFP+ to total DAPI+ cells. Values are mean + SEM, n = 9. MEF2CA, constitutively active MEF2C; IRES, internal ribosome entry site; MEF2 RE, MEF2 response element. (TIF) [file pone.0024027.s004.tif]

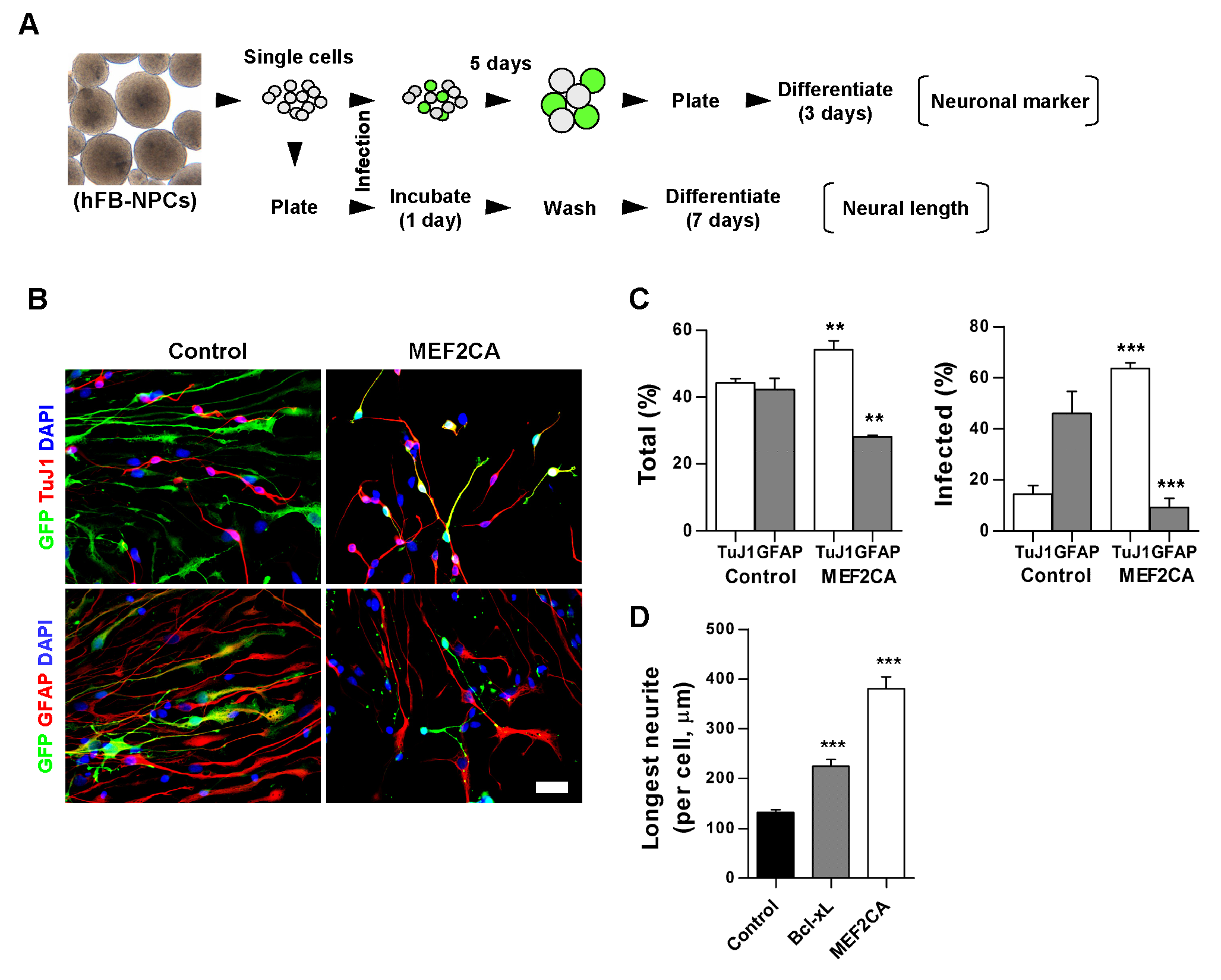

Supplement: Figure S4 — Neurogenic effect of MEF2CA on human fetal brain-derived neural progenitor cells (hFB-NPCs). (A) Schematic diagram showing the two differentiation procedures used here. (B) Fluorescent images of cells infected by lenti-control or -MEF2CA virus. For assessment of neuronal markers, hFB-NPCs were differentiated by the upper protocol shown in (A). Cells were double labeled with an anti-GFP to identify viral-infected cells and anti-TuJ1 to identify newly generated neurons, or with anti-GFAP to label neural precursor cells or astrocytes. Scale bar: 25 µm. (C) Quantification of fluorescent marker data after differentiation of control-infected and lenti-MEF2CA—infected cells. Plots show TuJ1+ and GFAP+ versus total cells (left), and TuJ1+ and GFAP+ versus GFP+/infected cells (right). Values are mean + SEM, n = 10; **p<0.01, ***p<0.001 by ANOVA. (D) The longest neuronal process per cell was measured with Neuron J software. Values are mean + SEM, n = 50 cells counted for each lentiviral infection; ***p<0.0001 compared to control by ANOVA. (TIF) [file pone.0024027.s005.tif]

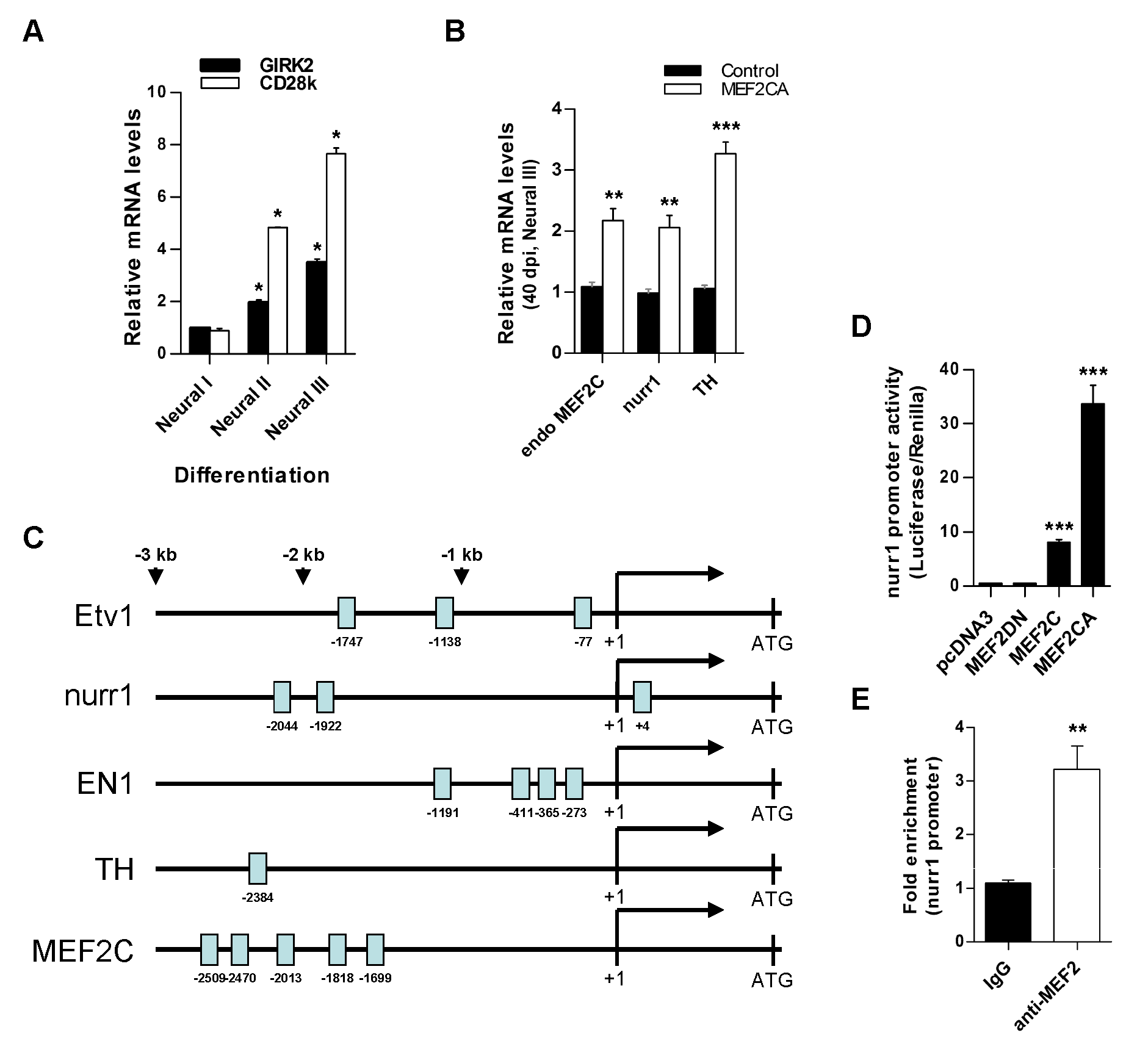

Supplement: Figure S5 — Enrichment of DA neuronal markers and promoter analysis of DA neuron-related genes in MEF2CA-infected cells derived from R-NSCs. (A) Relative mRNA levels of GIRK2 and CD28k were assessed throughout development in vitro. Values are mean + SEM, n = 3; *p<0.001 for values greater than in Neural Stage I by ANOVA. (B) Endogenous expression of MEF2C and nurr1 in MEF2CA-infected cells were analyzed by qPCR during Neural Stage III at 40 dpi. Values are mean + SEM, n = 3; **p<0.003, ***p<0.0002 compared to respective control by t-test. (C) Schematic diagram of putative MEF2 binding sites in the enhancer/promoter of various DA neuron-related genes. (D) Effects of various MEF2C constructs on nurr1 promoter activity. HeLa cells were cotransfected with empty vector, dominant negative MEF2C (MEF2DN), full-length wild-type MEF2C, or constitutively active MEF2C (MEF2CA) plus a nurr1 promoter (1.3 kb)-luciferase construct. Values are mean + SEM, n = 3; ***p<0.001 by ANOVA. (E) ChIP analysis of MEF2C association with the nurr1 promoter. After chromatin immunoprecipitation with anti-MEF2 antibody, qPCR primers detected the MEF2C response element in the nurr1 promoter region. Values are mean + SEM, n = 3; **p<0.01 by t-test. (TIF) [file pone.0024027.s006.tif]

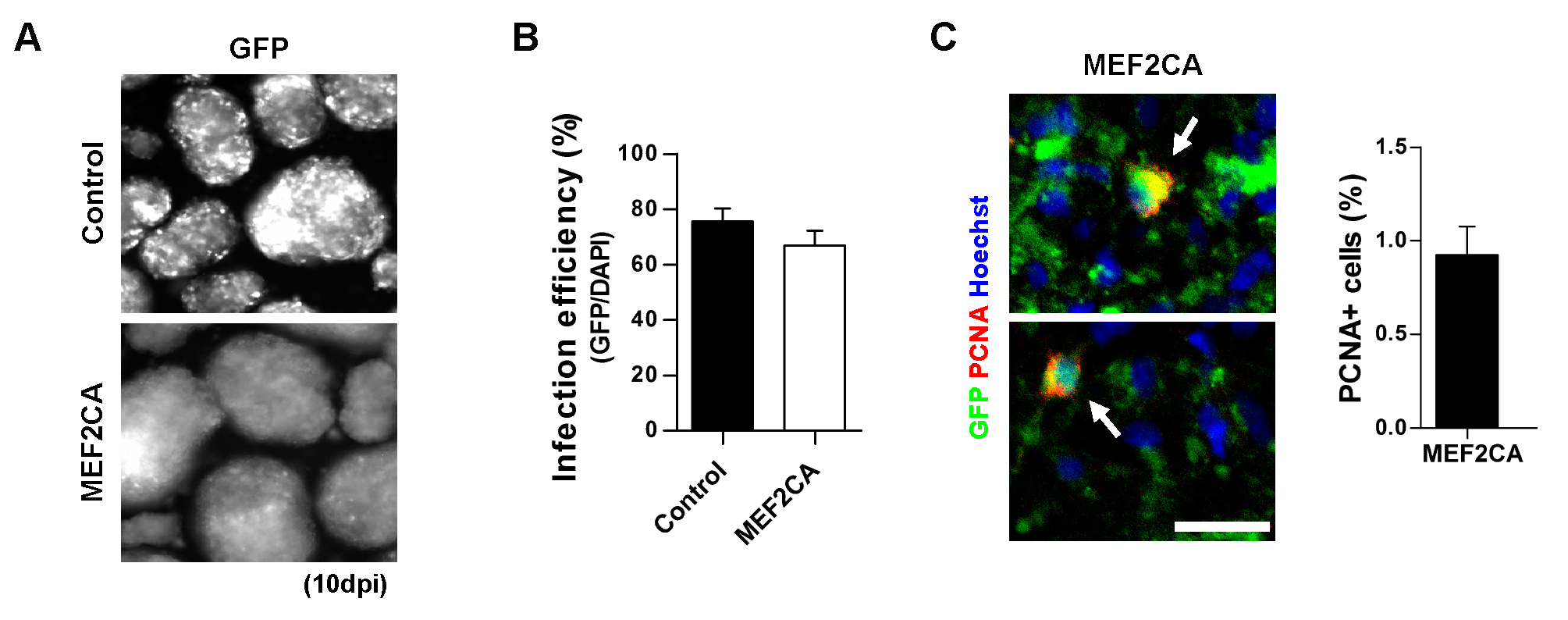

Supplement: Figure S6 — Infection efficiency of control-lentiviral vector and lenti-MEF2CA in hESC-derived R-NSCs. (A) Control- or MEF2CA-infected R-NSCs were grown as neurospheres for one week in preparation for their transplantation. Note that the MEF2CA construct bears an IRES, which results in weaker expression of GFP. (B) Infection efficiency of control-lentiviral vector and lenti-MEF2CA. To count infected cells, an aliquot of R-NSCs was plated onto poly-l-ornithine/laminin-coated coverslips and stained with anti-GFP antibody. Values are mean + SEM, n = 9. (C) Twelve weeks after transplantation, 0.9±0.15% of the engrafted MEF2CA/R-NSCs (green) expressed PCNA (red). Arrows indicate PCNA+ cells among transplanted cells; n = 13 experiments with 2,200 cells scored (quantified in histogram at right). Scale bar: 25 µm. (TIF) [file pone.0024027.s007.tif]

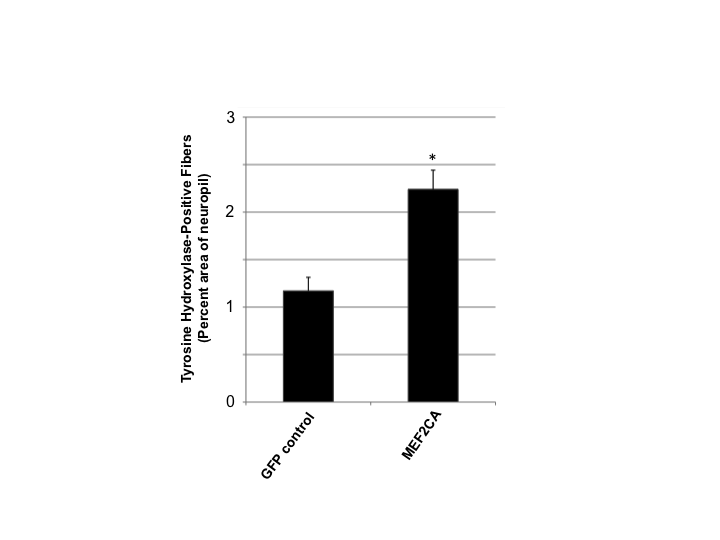

Supplement: Figure S7 — Increase in tyrosine hydroxylase-positive neuropil in host tissue adjacent to graft. Tissue sections were stained for tyrosine hydroxylase (TH), and the intensity of staining in the neuropil adjacent to the graft was measured. Random fields (n = 7 randomly chosen fields for each of 4 animals) were interrogated and adjusted for background staining by subtracting the intensity of a similar field distal to the graft. Values are mean + SEM, *p<0.0001 by t-test. (TIF) [file pone.0024027.s008.tif]
